# Mechanistic constraints in dengue severity: a systematic review with evidence stratification and agent-based evaluation of logical sufficiency

**DOI:** 10.3389/fimmu.2026.1831371

**Published:** 2026-06-19

**Authors:** Roberto Navarro Quiroz, Katherine Escorcia Lindo, Andrea Jaruffe Pinilla, Yiris Diaz-Olmos, Noelia Geribaldi-Dóldan, Cecilia Fernández-Ponce, Eloina Zarate Peñata, Yesit Bello Lemus, Lisandro Pacheco Lugo, Leonardo Pacheco Londoño, Antonio Acosta-Hoyos, Nataly Galan Freyle, Elkin Navarro Quiroz

**Affiliations:** 1 Center for Research in Critical Dynamics, Barranquilla, Colombia; 2 Tecnológico de Antioquia—Institución Universitaria, Medellín, Colombia; 3 Facultad de Ciencias Básicas y Biomédicas, Centro de Investigaciones en Ciencias de la Vida (CICV), Universidad Simón Bolívar, Barranquilla, Colombia; 4 División de Ciencias de la Salud, Programa de Medicina, Universidad del Norte, Barranquilla, Colombia; 5 Instituto de Investigación e Innovación Biomédica de Cádiz (INiBICA), Cádiz, Spain; 6 Departamento de Anatomía, Facultad de Medicina, Universidad de Cádiz, Cádiz, Spain; 7 Departamento de Biomedicina, Biotecnología y Salud Pública, Facultad de Medicina, Universidad de Cádiz, Cádiz, Spain

**Keywords:** agent-based model, dengue hemorrhagic fever, glycocalyx shedding, myeloid activation, NS1 protein, systematic review, vascular permeability

## Abstract

**Background:**

Severe dengue is a vascular immunopathology characterized by plasma leakage, thrombocytopenia, hemorrhage, and, in its most critical form, dengue shock syndrome. Although NS1-mediated endothelial injury, glycocalyx disruption, inflammatory myeloid activation, and coagulation/platelet abnormalities have all been implicated, it remains unclear which mechanisms are most consistently supported and whether they form a coherent functional architecture capable of explaining vascular decompensation. This review asks two linked questions: which dengue mechanisms are supported by the contemporary evidence base, and whether the strongest supported components are logically sufficient, when coupled, to generate a synthetic analog of connected endothelial-barrier failure.

**Methods:**

We conducted a PubMed-indexed systematic review of dengue mechanistic studies published from 2020 to 2025 under a dengue-only eligibility policy. Full texts were assigned to six mechanism families, graded on a five-tier evidence scale, and classified using predefined claim ceilings: C0 empirical restriction, C1_conditional regularity, or C2 exploratory evidence. Meta-analysis readiness was assessed using PICOS criteria. A constraint-first agent-based model (ABM) was then used as a permanent C2 logical sufficiency evaluator to test whether the three strongest evidence families could jointly generate a synthetic analogue of connected endothelial-barrier failure under explicit assumptions.

**Results:**

Of 200 retrieved records, 59 were included after full-text adjudication. Three mechanism families reached C1_conditional evidence: NS1-linked vascular permeability (DENV-M01, n=23), endothelial glycocalyx/barrier disruption (DENV-M02, n=17), and myeloid effector activation (DENV-M03, n=12). Receptor gating, coagulopathy/platelet dysregulation, and therapeutic mechanistic targets remained C2 evidence-gap families. Two null randomized trials imposed C0 restrictions: rupatadine did not significantly reduce plasma leakage (RR = 0.68, 95% CI 0.41–1.12), and oseltamivir did not improve time to defervescence (MD =+ 0.1 days, p=0.055). No mechanism family was eligible for quantitative pooling because CI-bearing estimates were sparse and outcome definitions were insufficiently harmonized. In the ABM, the review-supported NS1–barrier–myeloid set generated a spatially connected endothelial-barrier failure analog. This analog emerged when upstream viral/NS1 pressure and myeloid collateral cost exceeded barrier reserve and repair capacity. The regime remained stable under changes in update rule, rule form, and spatial patch scale, indicating that it was not a single implementation artefact. Boundary location was more stable than local execution timing, whereas high heterogeneity intensity produced only bounded boundary displacement. Minimality ablation showed partial, not complete, minimality: upstream pressure and barrier fragility were load-bearing, whereas the myeloid arm was phase-dependent and counter-directional, consistent with a dual role in early containment and late collateral damage within the model.

**Clinical trial registration:**

The current evidence supports a minimum-range organizational account of severe dengue vascular decompensation centered on the NS1–barrier–myeloid unit. This account is best interpreted as a competing-constraint model: viral/NS1 pressure, endothelial/glycocalyx barrier preservation, repair capacity, and myeloid effector control can become difficult to maintain within the same physiological window during progression toward vascular leakage. The ABM provides C2-level in silico support for logical sufficiency by showing that these review-supported components can generate a connected endothelial-barrier failure analog under explicit assumptions. It does not establish causal mechanistic validation, molecular equivalence, or patient-level prediction. Claim escalation now requires longitudinal cohorts measuring NS1/viraemia, endothelial barrier injury markers such as SDC1 or Ang-2, and myeloid effector proxies such as sTREM-1 or CXCL10, together with orthogonal functional perturbation assays reporting CI-bearing outcomes.

## 1 Introduction

### 1.1 Clinical burden and the mechanistic gap

Dengue virus (DENV) infects an estimated 400 million people annually and causes approximately 22,000 deaths, principally through dengue shock syndrome (DSS) characterized by rapid plasma leakage, thrombocytopenia, and hemorrhage (1–3). The clinical spectrum ranges from self-limiting febrile illness—the majority of infections—to a severe vascular phenotype that concentrates mortality risk in pediatric and immunocompromised populations (1). Despite this burden, the mechanistic transition from uncomplicated dengue to severe vascular immunopathology remains incompletely resolved within the recent recoverable dengue corpus. In particular, the molecular and cellular pathways that link DENV replication and NS1 secretion to endothelial barrier failure have not been systematically stratified by evidential strength or causal proximity across the 2020–2025 literature that currently drives mechanistic interpretation.

The absence of a formal evidence map generates a specific problem: mechanistic claims from individual studies migrate directly into review conclusions and grant narratives without stratification by reproducibility, study design, or logical coherence with null findings. This claim-level inflation impedes prioritization of experimental follow-up and conflates exploratory *in vitro* associations with reproducible clinical regularities (4). The present work addresses this gap using an explicit claim-stratification framework applied prospectively to a recoverable systematic evidence corpus. Its intended contribution is not an all-year recapitulation of dengue pathogenesis but a contemporary mechanistic audit of the 2020–2025 PubMed-indexed corpus that combines claim ceilings, null-RCT restrictions, meta-analysis feasibility assessment, and a governed review-to-model bridge within one auditable framework.

### 1.2 Dengue immunopathogenesis: from viremia to vascular collapse

Severe dengue is not primarily a consequence of viral load per se but of the host immune response to NS1 and its downstream inflammatory mediators (5, 6). DENV NS1 organizes intracellularly as a membrane-associated dimer and is predominantly secreted as a lipoprotein hexameric particle (7). Soluble NS1 binds to the endothelium and to myeloid cells, where it can disrupt the glycocalyx, trigger inflammatory signaling cascades, and promote vascular hyperpermeability through NS1-mediated endothelial stress (8–10). The evidential strength of these host–pathogen interaction pathways, however, is heterogeneous: some are supported by single *in vitro* studies; others by convergent clinical cohort, animal model, and mechanistic data.

A second layer of the mechanistic problem is the distinction between necessary and sufficient conditions. Even if NS1-mediated endothelial activation is an important upstream contributor to barrier failure and vascular leakage, it may not be sufficient in isolation: the literature consistently implicates a concurrent myeloid effector contribution—specifically, monocyte/macrophage inflammatory amplification producing cytokine-mediated collateral endothelial damage—as a required co-participant in the percolation-like vascular compromise observed in severe dengue. Whether these three components (viral/NS1 pressure, glycocalyx/barrier disruption, and myeloid effector activation) form a logically sufficient set has not been formally evaluated. The present work therefore treats these pathways as candidate contributors requiring formal evidential stratification rather than as established facts and uses their convergent directionality as the basis for a sufficiency test rather than as prior proof.

### 1.3 A restriction-first framework

We adopt a restriction-first epistemological stance: the viable mechanistic possibility space is first constrained by null interventional evidence before exploratory positive associations are interpreted. In this framework, C0 restrictions are established by negative RCTs or reproducible null findings that delimit mechanism space; C1_conditional claims are robust multi-study regularities; and C2 claims are exploratory, hypothesis-generating only. This ordering is motivated by the observation that null findings from rigorously conducted RCTs—negative results from rupatadine and oseltamivir trials—provide stronger constraints on the mechanism space than exploratory *in vitro* associations (11, 12). Positive mechanistic claims are evaluated against these restrictions before any synthesis conclusion is drawn.

In this framework, a mechanistic constraint is not treated as a universal law but as an empirically bounded condition that limits which pathogenic explanations remain viable. Severe dengue may be interpreted as a pathological transition arising when antiviral control, endothelial barrier preservation, and myeloid effector regulation become difficult to satisfy simultaneously within the same physiological window—a competing-constraint interpretation that organizes the synthesis without elevating any claim ceiling.

### 1.4 The NS1–barrier–myeloid organizational sufficiency theory

Based on convergent signals from the pre-review literature, we formulated before conducting the review a minimum-range organizational theory of severe dengue vascular decompensation. The theory proposes that the minimum functional set sufficient to generate a dengue barrier-collapse analog *in silico* consists of three concurrent components: (i) NS1-driven upstream endothelial pressure (DENV-M01/M02), (ii) glycocalyx/barrier disruption as a permissive state for vascular leakage (DENV-M02), and (iii) myeloid effector activation with collateral endothelial damage (DENV-M03). This is a testable sufficiency claim, not a claim of causal completeness, molecular specificity, or strict mechanistic necessity.

The theory is not a linear pathway model but a competing-constraint model in which antiviral pressure, endothelial barrier preservation, and myeloid effector control are treated as coupled biological demands whose incompatibility may generate a transition toward vascular decompensation.

The theory is asymmetric in the way a useful scientific theory should be. It would be weakened if the three-component regime failed to survive pre-declared falsification-oriented perturbations of update rules, rule forms, or spatial organization; it would also be weakened if the empirical restrictions from null dengue RCTs supported rival rate-limiting pathways. The systematic review and ABM were therefore designed not merely to collect recurrent signals but to test whether the recent corpus and a constraint-first computational analog support this minimum organizational sufficiency theory more strongly than single-component, downstream-only, or unrelated-signal explanations.

Three serious single-factor alternatives were considered and remain logically coherent within the corpus, but less parsimonious than the coupled theory: a viral-pressure-only account in which barrier and myeloid signals are downstream correlates, a barrier-first account in which NS1 and myeloid inputs act mainly as modifiers, and a myeloid-first account in which monocyte/macrophage inflammatory amplification is the primary organizer. A fourth alternative is that DENV-M01, DENV-M02, and DENV-M03 are recurrent but uncoupled signals rather than a functional module. The review addresses the first three alternatives through family-level evidence stratification and C0 restrictions, whereas the ABM addresses the fourth by testing whether the coupled regime survives multiple falsification-oriented probes under different computational assumptions.

### 1.5 Relationship to previous dengue mechanistic reviews

Previous dengue reviews have described NS1 biology, endothelial dysfunction, glycocalyx disruption, cytokine-mediated collateral damage, platelet and coagulation dysregulation, and the broader clinical pathogenesis of severe disease (5, 7, 13, 14). The present manuscript does not replace that literature and does not claim historical exhaustivity. Its narrower contribution is to ask which recent claim-bearing mechanistic records survive formal evidence stratification, how pre-2020 foundational citations should be separated from the 2020–2025 synthesis corpus, which C0 restrictions constrain therapeutic explanations, and whether the dominant review-derived module is logically sufficient in a bounded ABM without raising the causal claim ceiling.

### 1.6 Study objectives

The objectives of this work are (a) to characterize and stratify the dengue immunopathogenesis evidence landscape across six mechanism families using a formal claim framework; (b) to evaluate whether the proposed NS1–barrier–myeloid organizational sufficiency theory is logically sufficient to produce a synthetic barrier-collapse analog in a constraint-first agent-based model; and (c) to define the C0 restrictions and minimum viable datasets that govern future experimental and clinical claim escalation.

## 2 Materials and methods

### 2.1 Systematic review protocol

#### 2.1.1 Search strategy

PubMed was searched for dengue mechanistic studies published between January 2020 and December 2025. The search combined MeSH terms (Dengue/pathogenesis, Dengue Virus/pathogenicity, Vascular Permeability) with supplementary keyword strings targeting NS1, glycocalyx, heparanase, syndecan, TREM-1, myeloid activation, and severe dengue. This recency window was selected *a priori* to audit the contemporary mechanistic corpus that currently drives dengue interpretation and experimental prioritization; older foundational studies were retained as background citations but not treated as part of the claim-bearing synthesis set. Pre-2020 studies were cited only as foundational background and were not counted as claim-bearing synthesis units. The full search strategy is detailed in the supplementary protocol document. No language restrictions were applied to the search, but language barriers were documented as evidence-gap items in post-extraction audit.

#### 2.1.2 Eligibility criteria

Records were eligible for inclusion if they (i) reported original mechanistic data directly relevant to dengue immunopathogenesis; (ii) involved DENV infection, NS1 exposure, or dengue patient cohorts (non-dengue flaviviruses—YFV, JEV, OHFV, and WNV—were excluded under a dengue-only eligibility policy); and (iii) provided retrievable quantitative or direction-of-effect data. Review articles without original data were excluded. RCTs targeting dengue-relevant endpoints were included as C0 evidence regardless of mechanism family assignment.

#### 2.1.3 Screening and adjudication

Title and abstract screening followed by full-text retrieval were performed by two independent reviewers using a PRISMA 2020-compliant workflow (15). Discrepancies were resolved by consensus. Full-text records not accessible via open-access channels were recorded as evidence gaps; their mechanism family was not assigned. Records adjudicated after the primary search cycle (SCR-00074, SCR-00126, SCR-00146, and SCR-00154) were incorporated in a documented adjudication update (2026-03-11) before final synthesis. A final corpus-integrity refreeze (2026-03-14) removed non-primary and indirect or method-heavy records that failed primary-corpus adjudication and corrected multiple study-design miscategorizations before the submission freeze. All 59 claim-bearing included studies are listed individually in **Supplementary Table 1** with PMID/DOI, study design, population/model, mechanism family, outcome/domain, evidence tier, claim ceiling, reason for inclusion, and reference number.

#### 2.1.4 Evidence grading and claim classification

Each included record was assigned to one of six mechanism families and graded on a five-tier evidence scale:

- E1—single study, no replication
- E2—two or more studies with convergent direction, at least one non-*in vitro* design
- E3—multi-study convergence across ≥2 independent study designs (clinical cohort, *in vitro*, animal model)
- E4—functional mechanistic intervention with quantitative support
- E5—experimental causal (randomized, interventional, with CI-bearing dose-response)

Family-level claim ceilings were assigned as follows:

- C0—empirical restriction: established by negative RCT or reproducible null finding that delimits mechanism space
- C1_conditional—robust multi-study regularity (E3), context-dependent; does not imply causal sufficiency
- C2—exploratory: supported by E1 or E2 evidence; hypothesis-generating only

Claim ceilings are not promoted without CI-bearing quantitative evidence from an orthogonal experimental design. Claim governance was maintained prospectively throughout the project using a frozen evidence and wording framework.

#### 2.1.5 Meta-analysis eligibility

PICOS-based eligibility assessment for quantitative pooling was performed for the three C1_conditional families (DENV-M01, M02, and M03), following Cochrane Handbook Chapter 10 criteria (16). The PICOS definition and pooling eligibility criteria are summarized in **Table 1**. Pooling prerequisites required (i) ≥2 records within the same family reporting the same primary outcome metric; (ii) CIs available (non-FDR) for each contributing estimate; and (iii) sufficient design comparability for fixed- or random-effects estimation. Full PICOS audit results are reported in Section 3.9 and **Supplementary File S2**.

**Table 1:** Evidence summary by dengue mechanism family (n=59 included records).

PICOS domain	Operational definition
Population	Dengue patients, DENV/NS1-exposed systems, and dengue-relevant animal, organoid, or cell models; clinical records were required for quantitative pooling.
Intervention/exposure	DENV infection, NS1 exposure, mechanistic perturbation, or dengue-relevant therapeutic intervention.
Comparator	Non-severe dengue, dengue without warning signs, healthy controls, placebo, unexposed cells/animals, or baseline condition.
Outcomes	Vascular permeability, plasma leakage, glycocalyx or endothelial barrier injury, myeloid effector activation, platelet and coagulation dysregulation endpoints, and severity-linked quantitative outcomes.
Study designs	Clinical cohorts, cross-sectional studies, *in vitro* systems, animal models, organoid systems, and RCTs for C0 restrictions; pooling required comparable clinical designs.
Pooling criterion	At least two records with the same metric, explicit CI or SE, and comparable population/design; otherwise, narrative synthesis and meta-analysis readiness auditing were used.

### 2.2 Agent-based model—logical sufficiency evaluator

#### 2.2.1 Model architecture and rationale

A two-dimensional lattice agent-based model (ABM) was constructed to evaluate whether the three C1_conditional mechanisms—NS1-driven vascular pressure (DENV-M01), endothelial barrier disruption via glycocalyx shedding (DENV-M02), and myeloid effector activation with collateral damage (DENV-M03)—are jointly sufficient to produce a synthetic barrier-collapse analog in a constrained computational system. The ABM is not a molecular reconstruction of dengue immunopathogenesis; it is an organizational analog that captures functional roles at the coarse-grained level of (i) upstream infectious pressure; (ii) costly innate-effector amplification, defined here as myeloid effector cost representing inflammatory amplification and collateral endothelial damage associated with monocyte/macrophage activation; (iii) finite repair capacity; and (iv) spatially connected damage propagation.

The model operates on an NxN toroidal or bounded grid (default N = 18). Each node occupies one of four states: INTACT, INFECTED, DAMAGED, or REPAIRED. Three grids track continuous quantities per node: viralgrid (DENV-M01/M02 analog), immunegrid (DENV-M03 analog), and damagegrid. The primary outcome metric is spanning probability: the fraction of simulation replicates in which damaged nodes form a contiguous cluster connecting opposite edges of the grid—a synthetic analog of barrier-compromise propagation representing spatially connected endothelial-barrier compromise, not patient-level plasma leakage.

The model implements a phase-separated immune module with an early containment phase (time <12 steps) favoring viral clearance, and a late dysregulation phase (time ≥12 steps) decoupling recruitment gain from clearance efficiency and amplifying collateral damage. This phase separation instantiates the known temporal dynamics of dengue immunopathology—initial innate containment followed by immunopathological amplification in severe cases (13, 14).

#### 2.2.2 Parameters and calibration

Key free parameters include viralreplicationrate (VRR), recruitmentsensitivity (RecS), collateraldamagerate (CDR), repairrate, and maxmyeloiddensity. Parameters were not calibrated to specific clinical time-series data. Instead, they were explored as a regime-defining space: The claim of interest is whether a connected-failure regime exists, not where specific parameter values lie. All sweep boundaries were pre-registered before data generation. Parameter ranges are reported in full in the regime atlas (Section 3.7.1 and **Supplementary File S1**).

#### 2.2.3 U1 falsification-oriented battery and U2 structured enrichment

To test whether the organizational sufficiency claim could be falsified by ordinary modelling artefacts or hidden implementation dependence, we conducted a four-component pre-declared falsification-oriented battery (U1) before using ABM results in any interpretation. Each U1 component targeted a distinct alternative explanation for the apparent regime: synchrony dependence (U1-A), path-order dependence that would dissolve the coupled reading (U1-B), rule-form dependence (U1-C), or projection-specific artefact in the atlas views (U1-D):

- U1-A—update-rule sensitivity: synchronous (double-buffered) versus asynchronous (in-place, random-order) node update. Pre-declared verdict threshold: robust if max delta(spanning_probability) < 0.10 across all parameter sets.
- U1-B—hysteresis/path-dependence sweep: directional sweep of viral replication rate along a near-boundary track (ascending then descending), testing for finite-time path dependence.
- U1-C—rule-form perturbation: all eight combinations of linear versus saturating recruitment, binary versus graded repair, and linear versus saturating collateral damage. Pre-declared verdict threshold: robust if max delta <0.10 across all combinations.
- U1-D—regime atlas: one transition-resolving 2D phase map (VRR × RecS) and one execution-timing map (VDR × repair_rate) within a saturated connected-failure region (≥10×10 grid, ≥32 replicates per point).

Following U1, a six-component structured-enrichment battery (U2) was conducted within the transition zone. In this sense, U2 functions as a second layer of falsification-oriented probing under richer structural conditions:

- U2-A—clearance-collateral cost arm separation.
- U2-B—repair modulation.
- U2-C—barrier heterogeneity (uniform vs. patchy, fixed strength=0.50).
- U2-D—patch-scale dependence (patchscale 1–9 at fixed RecS=0.50; verdict: PATCHSCALE_ROBUST, max boundary shift=0.00).
- U2-E—cross-band RecS expansion (RecS 0.20–0.80, patchscales 2/4/6; verdict: RECSBOUNDARY_ROBUST, global max boundary shift=0.02).
- U2-F—heterogeneity-strength expansion (strength 0.10–0.90 × RecS 0.20–0.80 × patchscales 2/6; verdict: STRENGTHBOUNDARYBOUNDEDSENSITIVITY, global max boundary shift=0.05, max span range at uniform-boundary VRR = 0.92). The U2-F verdict is weaker than U2-D and U2-E: the transition location is not invariant under all synthetic heterogeneity perturbations, but the maximum displacement (0.05 VRR units) is smaller than the boundary-proximal execution variability (span range 0.92), which remains the dominant source of sensitivity in the tested range.

The ABM claim ceiling is permanently C2. No result from the U1 or U2 batteries authorizes promotion to C1 or above. All ABM findings must be interpreted as *in silico* analog observations, not empirical mechanistic claims or patient-level validation.

#### 2.2.4 Anchor matrix—review to model correspondence

An explicit review-to-model anchor matrix and a complementary match/mismatch matrix were constructed to define the permitted and blocked interpretive connections between systematic review mechanism families and ABM components. Anchoring was restricted to the organizational level: model components correspond to functional roles (upstream pressure, effector cost, and repair capacity), not to specific molecules (NS1, HPSE, and SDC1). The anchor matrix is reported as **Table 2**, and the match/mismatch logic is summarized in Discussion.

**Table 2:** Review-to-model anchor matrix.

ABM component	Review mechanism(s)	Allowed interpretation	Blocked interpretation
viral_grid	DENV-M01, DENV-M02	Stronger upstream pressure → stronger downstream pathological burden	Model captures real DENV/NS1 kinetics; claim of patient-equivalent viral dynamics
immune_grid	DENV-M03, partial DENV-M01	Stronger recruitment co-occurs with myeloid effector cost and trade-off	Primary-neutrophil or differentiated-macrophage equivalence
repair_rate	DENV-M02, partial DENV-M01	Recovery capacity modulates regime outcome	True glycocalyx restitution rates or clinical recovery time
collateraldamagerate	DENV-M03, partial DENV-M02	Stronger effector density co-occurs with collateral endothelial damage cost signals	Direct endothelial injury rate or cytokine concentration equivalence
damage_grid nodes	DENV-M01, DENV-M02	Progression from local perturbation to barrier-compromised spatial states	One-to-one correspondence to *in vivo* endothelial cell states
Spanning event	DENV-M01, M02, M03 (coupled)	Coupled pressure, myeloid effector cost, and impaired recovery are sufficient for synthetic barrier-collapse analog	Plasma leakage equivalence; shock prediction; molecule-specific inevitability claim

Anchoring restricted to organizational level. Allowed wording: “review-anchored organizational analog”; “qualitatively aligned with”; “is consistent with”. Blocked wording: “validated mechanism”; “calibrated to review data”; “confirms patient pathway”.

## 3 Results

### 3.1 Study selection

**Figure 1:**
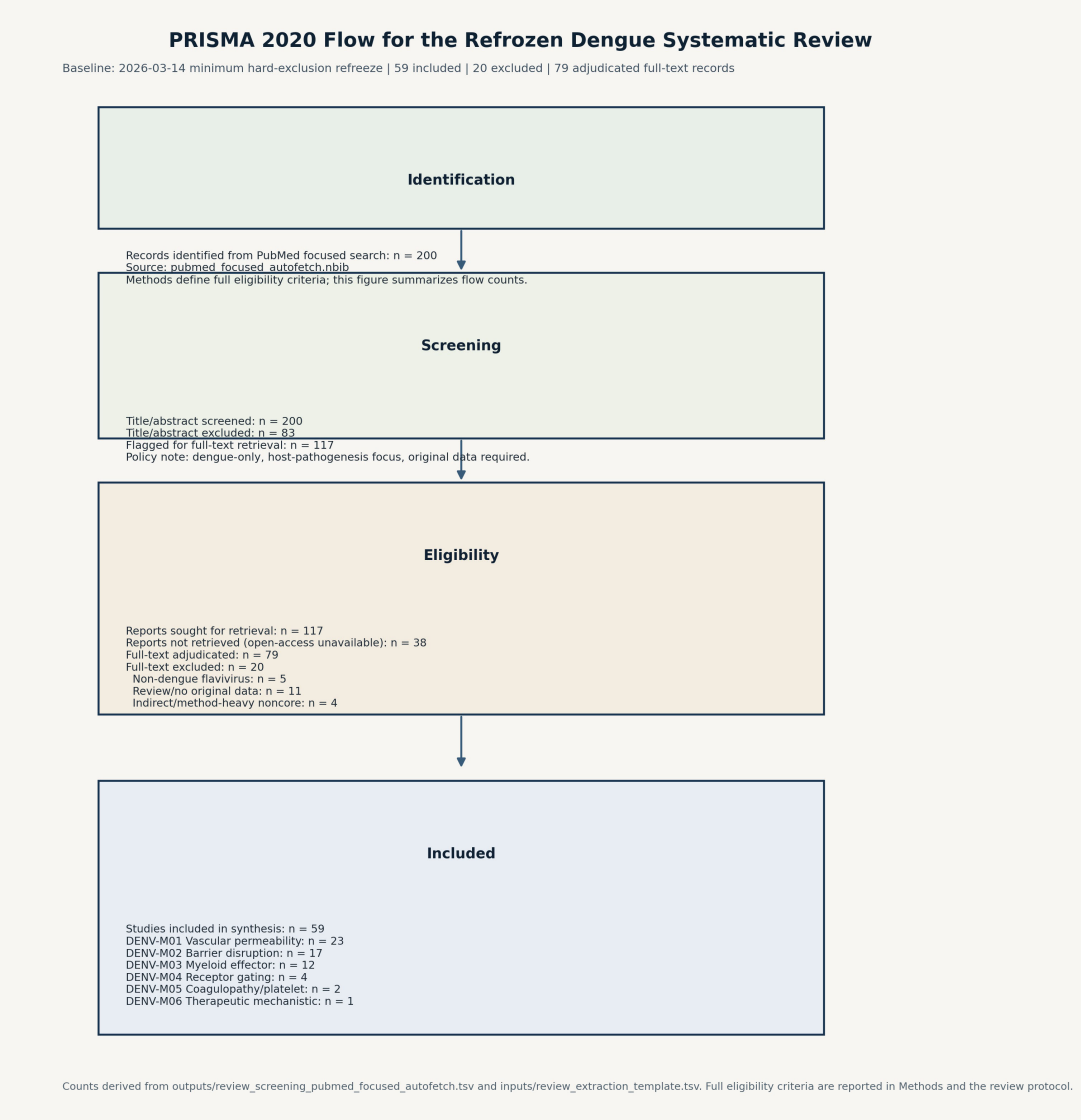
PRISMA 2020 flow diagram. The figure shows the full flow of the executed PubMed search from 200 retrieved records to 59 claim-bearing included studies. Records were excluded at title/abstract screening (n=83), flagged for full-text retrieval (n=117), not retrieved because open-access full text was unavailable (n=38), adjudicated at full text (n=79), and excluded after full-text review (n=20): non-dengue flavivirus (n=5), review/correction without original primary data (n=11), and indirect/method-heavy exploratory records removed from the primary convergence corpus (n=4). The source counts are provided in **Supplementary File S2** and **Supplementary Table 2**. No claim ceiling is assigned to the PRISMA flow itself.

A total of 200 records were retrieved from the PubMed targeted search. Following title and abstract screening, 83 records were excluded at the first stage. Of the 117 records flagged for full-text retrieval, 38 were not accessible via open-access channels and were recorded as evidence-gap items. Full-text adjudication was completed for 79 records. A total of 20 records were excluded at full text: 5 were non-dengue flavivirus records (SCR-00062, SCR-00102: YFV; SCR-00082: JEV; SCR-00034: OHFV; SCR-00171: WNV) excluded under the dengue-only eligibility policy, 11 were review or correction records without original primary mechanistic data, and 4 were original but indirect or method-heavy records removed from the primary convergence corpus during the final corpus-integrity refreeze. A total of 59 records were included in the final evidence synthesis (**Figure 1** — PRISMA 2020 flow diagram).

All synthesis counts and claim ceilings reported here reflect the final corpus freeze used for manuscript generation. All 59 claim-bearing included studies are listed individually in **Supplementary Table 1** and are included in the reference list; pre-2020 foundational references are cited separately as background and are not counted in the 2020–2025 synthesis denominator.

### 3.2 DENV-M01—NS1-dependent vascular permeability (C1_conditional, E3, n=23)

A total of 23 records addressed vascular permeability endpoints across the clinical cohort, *in vitro*, animal, and exploratory computational designs (17–35). Consistent with the C1_conditional classification, the directional signal was upward in 22/23 records: circulating NS1 and downstream mediators—IL-6, TNF-alpha, and ANGPTL4—were associated with increased endothelial permeability in severe dengue compared with non-severe dengue or healthy controls. These findings are consistent with NS1-mediated endothelial stress as an upstream driver of vascular leakage.

The most complete quantitative panel was reported in a serum-signature cohort study (SCR-00146) (36), which documented ROC-based discriminative performance for multiple biomarkers of vascular compromise: Ang-2 (AUC = 0.97; sensitivity 87.5%; specificity 90%), VEGF (AUC = 0.99; sensitivity 100%; specificity 98.6%), CRP (AUC = 0.92), and AST (AUC = 0.99). No explicit 95% CI or multivariable-adjusted model was recoverable from this record, precluding E4 promotion. The ROC panel AUC = 0.784 (95% CI 0.725–0.835; SCR-00015) (6) represents the strongest CI-bearing estimate available in this family and serves as the L2 anchor for meta-analysis readiness assessment.

C0 Restrictions. Two null RCTs within this family constitute empirical restrictions:

- Rupatadine (combined H1-antihistamine and platelet-activating factor antagonist): RR = 0.68 (95% CI 0.41–1.12; p=0.09) for the primary endpoint of plasma leakage reduction (11). This non-significant result indicates that the histamine/PAF axis is not rate-limiting in NS1-mediated vascular permeability.
- Oseltamivir (neuraminidase inhibitor): MD =+ 0.1 day (p=0.055) for time to defervescence (12). This result demonstrates no benefit from neuraminidase inhibition, distinguishing dengue barrier disruption from influenza sialic-acid-dependent mechanisms.

Evidence tier: E3. Claim ceiling: C1conditional. Promotion to C1mechanistic (E4) requires ≥1 CI-bearing quantitative outcome from an orthogonal experimental design.

### 3.3 DENV-M02—endothelial barrier disruption via glycocalyx shedding (C1_conditional, E3, n=17)

A total of 17 records documented NS1-mediated endothelial barrier dysfunction, converging on a glycocalyx-shedding axis (37–51). This family is mechanistically central to the coupled reading but quantitatively thinner than DENV-M01 and DENV-M03 because no CI-bearing estimate survives into pooling eligibility at the current evidence floor. Key signals across independent study designs included elevated heparanase (HPSE) activity consistent with glycocalyx disruption, reduced syndecan-1 (SDC1) and syndecan-4 (SDC4) surface expression, ANGPTL4 induction, DENV NS1-associated MMP-9 proteolytic activity linked to junctional damage, and endothelial tight-junction disruption in DENV-infected and NS1-exposed endothelial cell models. The DENV NS1 GDI-motif (residues 91–93) was identified as structurally required for vascular leak induction in a key structural-functional study (SCR-00126) (52). Anti-NS1 monoclonal antibody treatment preserved transendothelial electrical resistance (TEER) in HUVEC assay models (53), providing functional mechanistic support for the NS1-mediated endothelial stress axis.

Critical taxonomic note. The ERA mechanism ledger (a parallel evidence base for ARVI/long-COVID datasets used in this research program) designates a mechanism M03 involving glycocalyx shedding driven by influenza NS1—a distinct protein functioning as an interferon antagonist, not an endothelial disruptor. DENV-M02 (dengue NS1 + HPSE + SDC1/4 axis) and ERA-M03 (influenza NS1 interferon antagonism) are mechanistically non-overlapping despite the shared gene name. The dengue-only eligibility policy applied throughout this review ensures that no ARVI-context records contaminate this family.

Evidence tier: E3. Claim ceiling: C1_conditional.

### 3.4 DENV-M03—myeloid effector activation and cost (C1_conditional, E3, n=12)

A total of 12 records documented macrophage and monocyte inflammatory amplification associated with severe dengue (54–64). Cytokines IL-6, TNF-alpha, IL-10, and CXCL10 were reproducibly elevated in severe versus non-severe dengue across clinical cohort designs. Soluble TREM-1 (sTREM-1), a marker of myeloid effector activation reflecting monocyte/macrophage inflammatory state, was associated with severe dengue with OR = 3.8 (95% CI 1.6–10) in the single available multivariable-adjusted study (SCR-00037) (65). This constitutes the strongest CI-bearing mechanistic estimate in the entire corpus and serves as the L2 anchor for DENV-M03 meta-analysis readiness. AXL receptor-mediated monocyte tropism was identified as a dengue entry and activation route, linking DENV infection to myeloid inflammatory amplification.

Mechanistic integration. DENV-M01, M02, and M03 can be read as functionally coupled at the level of cross-study synthesis. NS1-mediated glycocalyx shedding (DENV-M02) is consistent with amplified myeloid effector output through loss of proteoglycan-dependent cytokine sequestration, which in turn could potentiate downstream vascular permeability (DENV-M01). This integration identifies the strongest organizational pattern in the current corpus, but it remains a synthesis-level interpretation—a review-anchored organizational analog—rather than a directly observed triad within a single cohort or perturbation study.

Evidence tier: E3. Claim ceiling: C1_conditional.

### 3.5 Exploratory mechanisms (C2 evidence-gap families)

DENV-M04, DENV-M05, and DENV-M06 are reported as low-density exploratory evidence-gap families, not as co-equal supports for the NS1–barrier–myeloid organizational theory. Their purpose in the synthesis is to preserve traceability and define replication needs while preventing sparse signals from being silently promoted.

DENV-M04—receptor gating (n=4, E2, C2). Four records addressed receptor-level entry and activation mechanisms (66–68). Vimentin was identified as a surface receptor facilitating NS1 endocytosis. TLR2/TLR6 heterodimer activation via NS1 engaged NF-kB-dependent inflammatory signaling. SR-B1/HDL-mediated context gating was supported by one record (SCR-00154), whereas NS1–HDL docking and pro-inflammatory lipoprotein reprogramming added a second receptor-context record (SCR-00140) (69). Cross-study replication remains insufficient for C1 promotion. At least two independent studies with convergent direction, including ≥1 non-*in vitro* design, are required before claim elevation.

DENV-M05—coagulopathy and platelet dysregulation (n=2, E2, C2). Two records documented platelet-mediated coagulopathy (70). Recombinant DENV-3 domain III (rEIII-Ig) reduced platelet activation *in vitro*. Caspase-3 activation was documented in dengue-associated thrombocytopenia. This remains an exploratory evidence gap with insufficient density for quantitative analysis or C1 claim.

DENV-M06—therapeutic mechanistic targets (n=1, E1, C2). One record addressed the eNOS/NO pathway: bradykinin combined with L-NMMA targeting vascular tone normalization. This single-study family has the lowest evidence density in the corpus and is treated only as an evidence gap; it cannot support any therapeutic mechanistic claim at this baseline.

### 3.6 C0 restrictions: null RCT evidence and the therapeutic constraint space

The two negative RCT findings are reported as full-section C0 restrictions because they constitute positive information about the mechanism space—they define what cannot occur—rather than merely negative trial outcomes. In this manuscript, C0 operates as an interpretive restriction category derived from null interventional evidence; it does not replace the row-level family ceiling assignments used in **Supplementary Table 1**.

Rupatadine (H1/PAF antagonist): in a randomized, double-blind trial targeting plasma leakage reduction in dengue fever, rupatadine failed to reach significance (RR = 0.68, 95% CI 0.41–1.12; p=0.09) (11). The CI does not exclude moderate benefit, but the point estimate is insufficient to support a mechanistic claim for histamine/PAF as a rate-limiting step in NS1-mediated vascular permeability. Any future intervention targeting this axis must account for this null finding.

Oseltamivir (neuraminidase inhibitor): in a randomized trial for dengue, oseltamivir showed no significant effect on time to defervescence (MD =+ 0.1 day; p=0.055) and no modification of vascular endpoints (12). This result distinguishes dengue barrier disruption from influenza sialic acid-dependent mechanisms and excludes neuraminidase inhibition as a dengue vascular pathway.

These restrictions are mechanistically informative: both null results are compatible with the NS1–HPSE–SDC1/4 axis that constitutes DENV-M02, which does not depend on histamine or neuraminidase activity.

### 3.7 Agent-based model—logical sufficiency evaluation

#### 3.7.1 Regime atlas (U1-D)

Two phase maps were generated across pre-registered parameter ranges to characterize the topology of the connected-failure regime.

**Figure 2:**
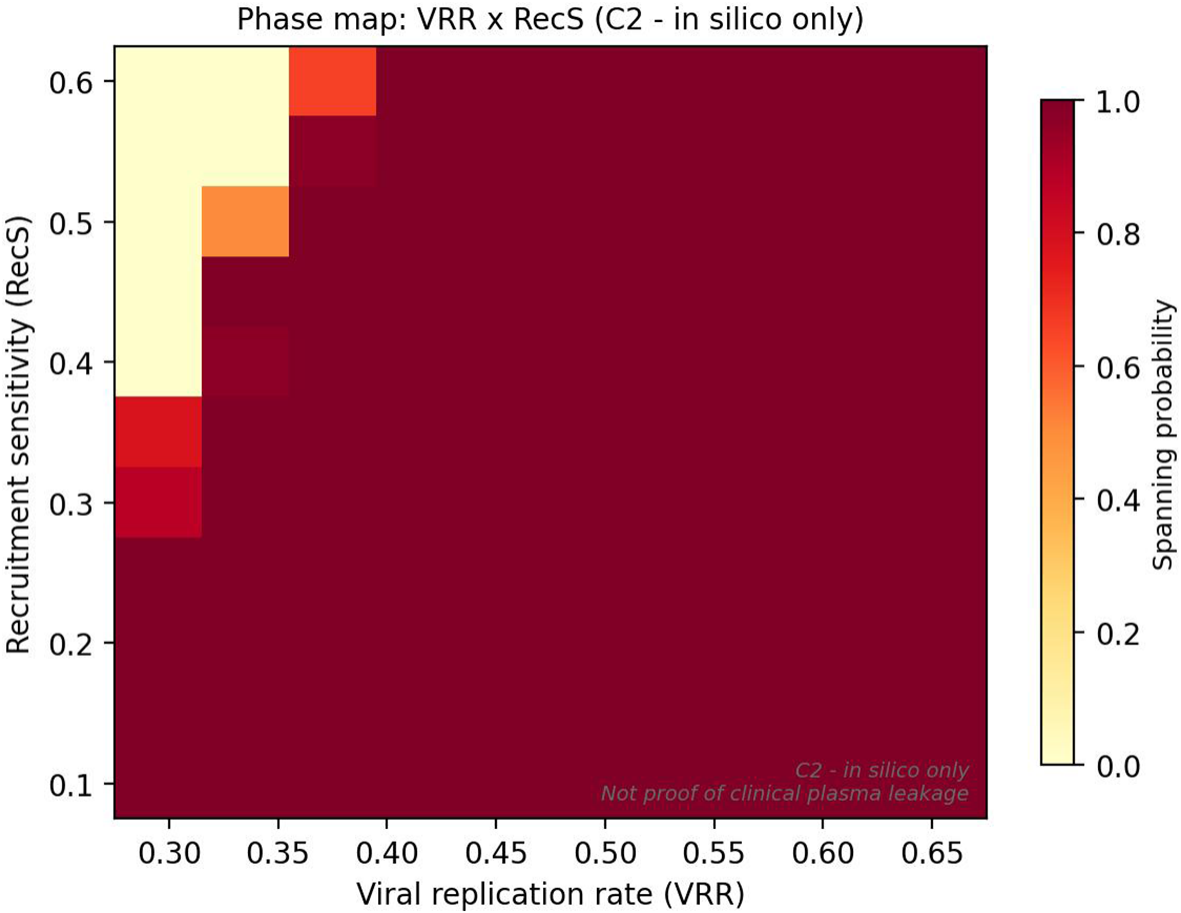
Phase Map 1: spanning probability across viral replication rate (VRR, x-axis) and immune recruitment sensitivity (RecS, y-axis). Each cell summarizes 32 ABM replicates from the VRR × RecS sweep in **Supplementary File S1**. Darker cells indicate a higher fraction of simulations in which damaged nodes formed a connected spanning cluster. Transitional points occur across VRR = 0.30–0.38, with RecS values spanning 0.30–0.60 in the resolved boundary band. The figure visualizes the C2 *in silico* transition boundary and does not constitute patient-level prediction or molecular validation.

Phase Map 1—VRR × RecS (Viral Replication Rate × Immune Recruitment Sensitivity): A 10×11 grid (110 parameter points; 32 replicates per point; 3,520 model runs total) was swept across VRR in [0.30, 0.65] and RecS in [0.10, 0.60]. Results (**Figure 2**):

- No-spanning regime (spanning_probability = 0): 7/110 points (6.4%), located in the low-VRR, high-RecS corner (VRR 0.30–0.35, RecS 0.40–0.60).
- Transitional regime (0 < spanning_probability < 1): 6/110 points (5.5%), forming a narrow slanted boundary band across VRR 0.30–0.38 and RecS 0.30–0.60.
- Saturated-spanning regime (spanning_probability = 1): 97/110 points (88.2%), covering the majority of the explored parameter space.

The phase boundary is sharp: the transition from no-spanning to full-spanning occurs within approximately 0.08 VRR units across the resolved boundary track. The sharpness indicates that the regime is robustly established above threshold and not a gradual drift—consistent with a genuine phase transition rather than a smooth interpolation.

**Figure 3:**
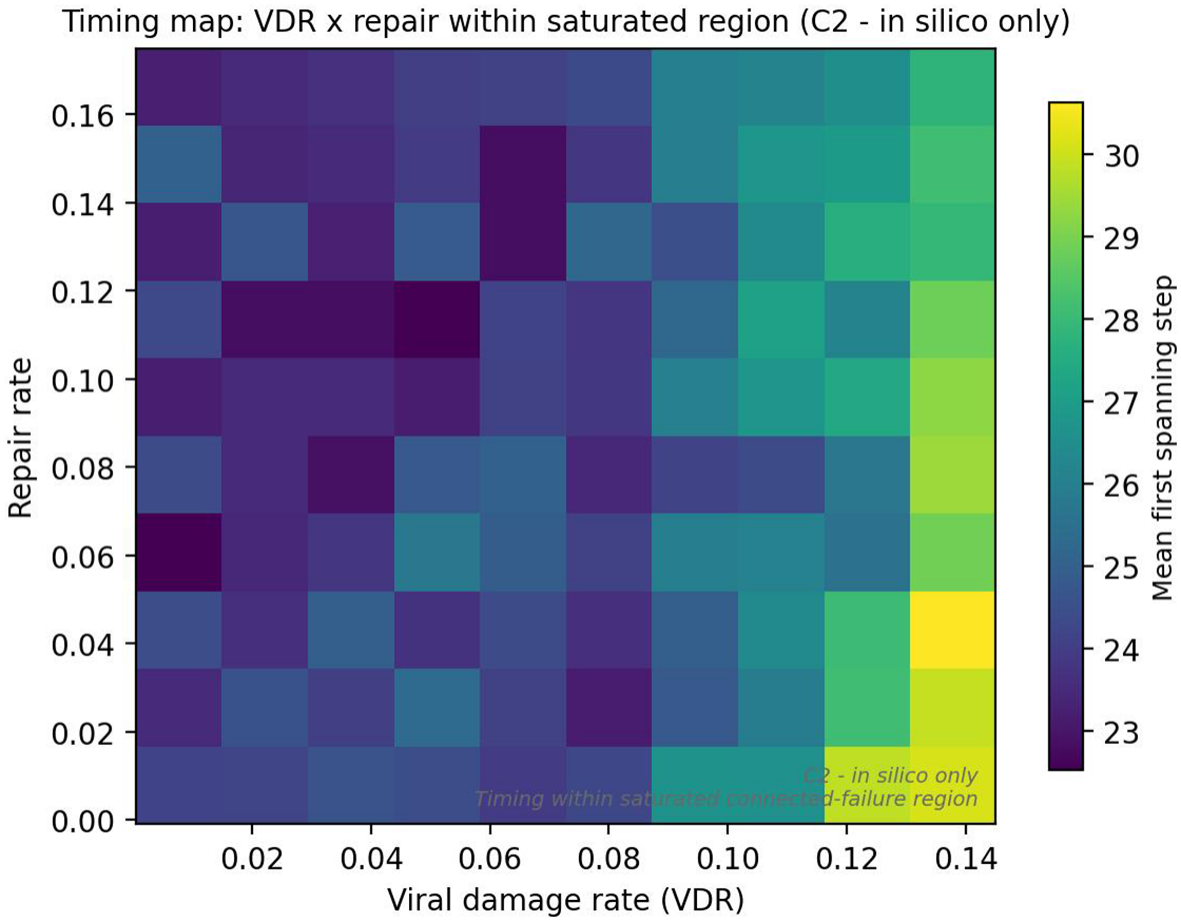
Execution-timing map across viral damage rate (VDR, x-axis) and cellular repair rate (y-axis) under fixed upstream pressure (VRR = 0.45, RecS=0.55). All 100 parameter points remain in the saturated connected-failure regime; color therefore represents mean first spanning step rather than spanning probability. Source data are provided in **Supplementary File S1**. The claim ceiling is C2, and the panel is interpreted as execution timing within the synthetic model, not as a second clinical phase-boundary map.

Phase Map 2—VDR × repairrate (Viral Damage Rate × Cellular Repair Rate): A 10×10 grid (100 parameter points; 32 replicates per point; 3,200 model runs total) was swept across VDR in [0.002, 0.130] and repairrate in [0.001, 0.160]. Results (**Figure 3**): all 100 parameter points showed spanning probability = 1.0, so this panel is not interpreted as a second transition-resolving map. Instead, it functions as an execution-timing map inside an already saturated connected-failure region under the fixed upstream parameters (VRR = 0.45, RecS=0.55). The mean first spanning step ranged from 22.53 to 30.63 across the grid. The gradient is directional: higher VDR values accelerate the onset of connected failure (shorter mean first spanning step), whereas repairrate exerts weaker and less monotonic modulation across the explored range.

#### 3.7.2 U1 robustness battery results and U2 structured enrichment

Four robustness checks were completed before any interpretive claim was made from ABM outputs. Results are summarized in **Table 3**.

**Table 3:** Evidence summary by dengue mechanism family (n=59 included records).

Mechanism family	ID	n records	Study designs	E-tier	Claim ceiling	Key signal	Key constraint
NS1-dependent vascular permeability	DENV-M01	23	Clinical, *in vitro*, animal	E3	C1_conditional	IL-6, TNF, ANGPTL4; Ang-2 AUC = 0.97	2 null RCTs (rupatadine, oseltamivir)
Glycocalyx-barrier disruption	DENV-M02	17	Clinical, *in vitro*, structural	E3	C1_conditional	HPSE up, SDC1 down, SDC4 down; NS1 GDI-motif	NS1 context (DENV only, not influenza)
Myeloid effector activation	DENV-M03	12	Clinical cohort, *in vitro*	E3	C1_conditional	sTREM-1 OR = 3.8 (95% CI 1.6–10); AXL	Single multivariable-adjusted study
Receptor gating	DENV-M04	4	*In vitro*, structural	E2	C2	Vimentin, TLR2/TLR6, SR-B1/HDL, NS1-HDL docking	No non-*in vitro* replication
Coagulopathy/platelet dysregulation	DENV-M05	2	Clinical, animal	E2	C2	rEIII-Ig, caspase-3	Insufficient for pooling
Therapeutic targets	DENV-M06	1	Organoid	E1	C2	eNOS/NO (bradykinin + L-NMMA)	Single study, no replication

E3 = multi-study convergence across ≥2 independent study designs. C1_conditional = robust replicated regularity, context-dependent; does not imply causal sufficiency. C2 = exploratory. C0 = empirical restriction (null findings).

U1-A—Update-Rule Sensitivity (verdict: UPDATE-RULE ROBUST). Synchronous (double-buffered) and asynchronous (in-place, random-order) node update modes were compared across six calibrated parameter sets and a 5×5 phase map (992 total model runs). Maximum delta(spanning_probability) = 0.000 across all 31 tested parameter sets. The connected-failure regime does not depend on global synchronization of node updates; it is an emergent structural property of the model dynamics, not an implementation artefact.

U1-B—Hysteresis/Path-Dependence (verdict: PATHDEPENDENT). A directional sweep of viral replication rate along a near-boundary track (VRR ascending from 0.12 to 0.26, then descending) was conducted. Maximum |delta(spanningprobability)| = 0.938; maximum |delta(maxdamagedfraction)| = 0.645. Finite-time path dependence was confirmed: the model exhibits trajectory-dependent outcomes inside the transition zone. This is an *in silico* property—it does not constitute evidence of clinical hysteresis in dengue patients.

U1-C—Rule-Form Perturbation (verdict: FORM-RULE ROBUST). All eight combinations of functional forms—linear versus Michaelis–Menten saturating recruitment, binary versus graded repair, linear versus density-saturating collateral damage—were compared across the rank-1 calibrated configuration (1,056 total runs). Maximum delta(spanning_probability) = 0.000 across all tested combinations. The regime is not a consequence of the linear functional form chosen for the default model rules.

U1-D—Regime Atlas. See Section 3.7.1.

U2-A–U2-C (clearance-collateral separation, repair modulation, barrier heterogeneity). The connected-failure regime persisted under immune-arm disaggregation, repair suppression, and introduction of spatially patchy barrier heterogeneity (fixed strength=0.50).

U2-D—Patch-scale dependence (verdict: PATCHSCALEROBUST). Six patch sizes (1–9) were swept against seven VRR values at RecS=0.50 with 32 replicates per condition. Maximum absolute boundary shift: 0.000. Boundary location was fully insensitive to patch size; boundary-proximal execution varied substantially (range=0.59 at VRR = 0.35).

U2-E—Cross-band RecS expansion (verdict: RECSBOUNDARYROBUST). Patch-scale robustness was extended to five RecS bands (0.20, 0.35, 0.50, 0.65, 0.80) with patch scales 2, 4, and 6. Global maximum boundary shift: 0.02. Boundary location is more stable than boundary-proximal execution across the full recruitment range tested.

U2-F—Heterogeneity-strength expansion (verdict: STRENGTHBOUNDARYBOUNDED_SENSITIVITY). Five heterogeneity strengths (0.10–0.90) were combined with five RecS bands and two patch scales across seven VRR values and 12 replicates per condition. Global maximum boundary shift: 0.05. Maximum span range at the uniform-boundary VRR: 0.92. The U2-F verdict is weaker than U2-D and U2-E: boundary location has bounded sensitivity to heterogeneity intensity when strength reaches 0.90, but the displacement (0.05 VRR units) remains smaller than local execution variability (span range 0.92).

#### 3.7.3 Review-to-model anchor matrix

**Table 4** defines the permitted and blocked correspondences between systematic review mechanism families and ABM components. The anchoring principle is organizational: model components correspond to functional roles in the pathological cascade, not to specific molecules. No ABM component is claimed to directly represent NS1 secretion, HPSE activity, or SDC1 shedding at the molecular level.

**Table 4:** U1 robustness battery and U2 structured enrichment summary.

Test	Component	Verdict	Key metric	Total model runs	Claim implication
U1-A	Update-rule sensitivity	UPDATE-RULE ROBUST	max deltaspan=0.000	992	Regime not an update-order artefact
U1-B	Hysteresis/path-dependence	PATH_DEPENDENT	max deltaspan=0.938	~480	Trajectory-dependent outcomes in transition zone; not clinical hysteresis
U1-C	Rule-form perturbation	FORM-RULE ROBUST	max deltaspan=0.000	1,056	Regime not a consequence of linear functional form
U1-D	Regime atlas (2D phase maps)	PHASE TRANSITION RESOLVED	—	6,720	Phase boundary characterized (VRR×RecS); downstream map saturated
U2-D	Patch-scale dependence	PATCHSCALEROBUST	max boundary shift=0.000; near-boundary span range=0.59	~1,568	Boundary location independent of spatial grain at RecS=0.50; execution modulated
U2-E	Cross-band RecS expansion	RECSBOUNDARYROBUST	global max boundary shift=0.020	~4,480	Boundary location stable across RecS 0.20–0.80; small shift at RecS≥0.65 only
U2-F	Heterogeneity-strength expansion	STRENGTHBOUNDARYBOUNDED_SENSITIVITY	max boundary shift=0.050; max span range=0.92	4,620	Bounded sensitivity to strength; location not invariant at very high heterogeneity; execution variability dominates

All verdicts evaluated against pre-declared thresholds (material shift threshold: 0.03 VRR units for U2-D/E; 0.05 for U2-F boundary proxy). ABM claim ceiling: C2 (permanent). No result in this table authorizes mechanistic or clinical claim escalation.

### 3.8 Mechanism family distribution

**Figure 4:**
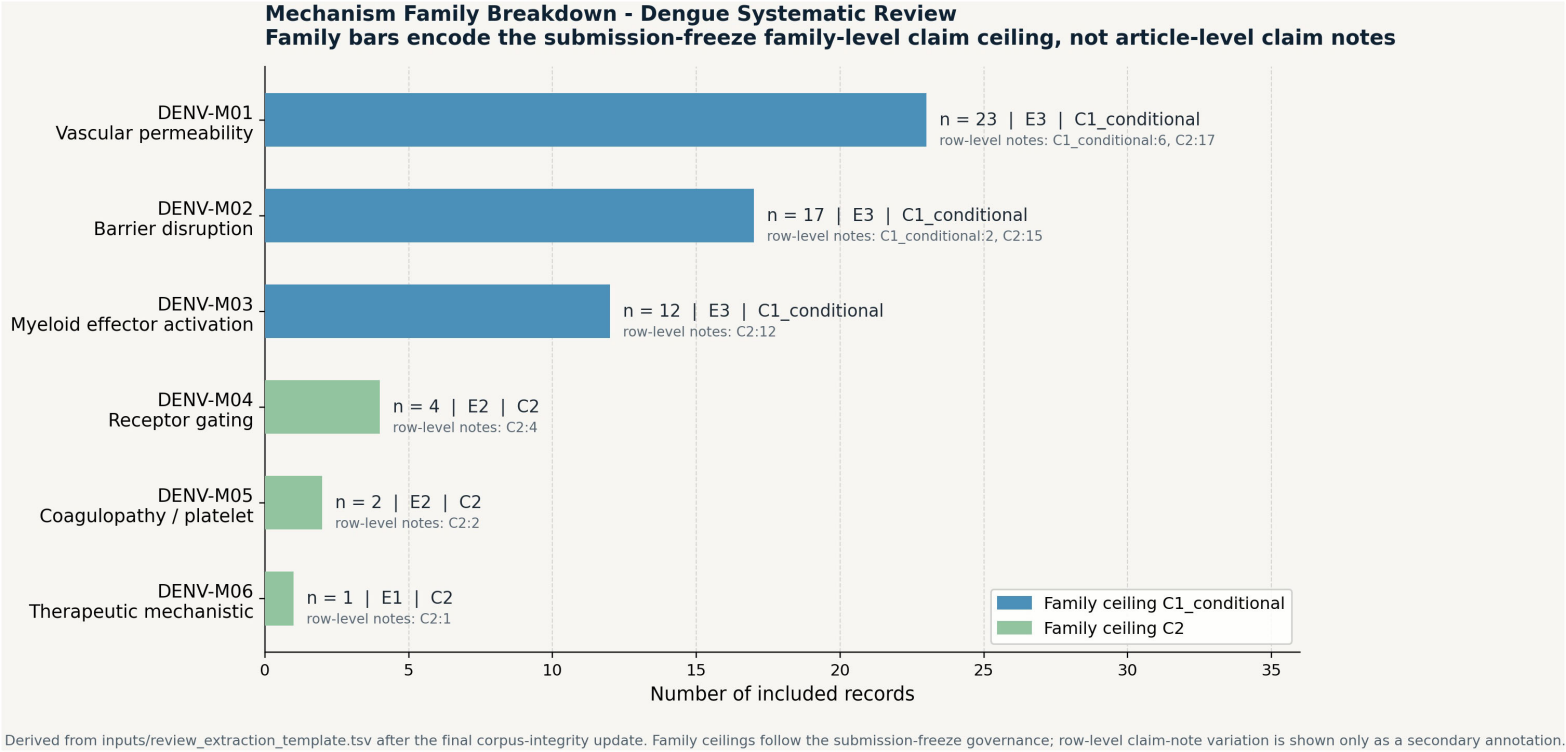
Mechanism family breakdown at the submission freeze. The horizontal bar chart shows the 59 claim-bearing included studies from **Supplementary Table 1** distributed across DENV-M01 to DENV-M06 and color-coded by family-level claim ceiling. DENV-M01 to DENV-M03 are the only C1_conditional families; DENV-M04 to DENV-M06 are low-density C2 evidence-gap families. The figure visualizes evidence density and ceiling assignment rather than effect size or causal strength.

The 59 included records distributed across six mechanism families as follows: DENV-M01 (n=23, 39.0%), DENV-M02 (n=17, 28.8%), DENV-M03 (n=12, 20.3%), DENV-M04 (n=4, 6.8%), DENV-M05 (n=2, 3.4%), DENV-M06 (n=1, 1.7%). The three C1_conditional families (DENV-M01–M03) account for 88.1% of the included record total (52/59; **Figure 4**—mechanism breakdown bar chart). The C2 families account for the remaining 11.9% and are concentrated in the receptor-gating and platelet-dysregulation categories.

### 3.9 Meta-analysis eligibility assessment

Formal PICOS-based eligibility assessment was performed for DENV-M01, M02, and M03. Quantitative pooling is not currently feasible for any of the three families. CI-bearing (non-FDR) estimates remain sparse: 4/23 records in M01 (17%), 0/17 in M02 (0%), and 1/12 in M03 (8%). No two records within any family share the same primary outcome metric with an available CI—the minimum criterion for a two-study fixed-effect pool (synthesis feasibility ladder L3). The current evidence base supports L2 (point estimate reporting with single CI) for DENV-M01 and DENV-M03 only: sTREM-1 OR = 3.8 (95% CI 1.6–10; SCR-00037) (65) for DENV-M03, and ROC-panel AUC = 0.784 (95% CI 0.725–0.835; SCR-00015) (6) for DENV-M01. DENV-M02 currently remains below L2 because no retained record contributes a CI-bearing barrier-disruption estimate.

The predominant reporting mode across the corpus is p-value without effect size or CI, FDR-corrected omics statistics (not poolable with clinical estimates), and qualitative directional statements. This reflects the current state of primary reporting in dengue mechanistic studies and is not a search limitation. Narrative synthesis, based on consistent upward directionality across 22/23 (M01), 15/17 (M02), and 12/12 (M03) records, is the methodologically appropriate approach given this evidence base and constitutes the empirical basis for E3 classification.

The minimum viable dataset for a first two-study pool in each family is (a) DENV-M01—one independent cohort reporting Ang-2 AUC with explicit 95% CI; (b) DENV-M02—extraction of CI from SCR-00052 ANGPTL4 OR plus independent cohort replication; (c) DENV-M03—one independent cohort replicating sTREM-1 ≥130 pg/mL OR with CI, which is the most achievable next milestone in the entire corpus.

## 4 Discussion

### 4.1 The NS1–barrier–myeloid module as a functional unit

The convergence of evidence across DENV-M01, M02, and M03—spanning clinical cohort, *in vitro*, structural, and animal model designs—supports interpreting them as the strongest candidate minimum organizational set in the contemporary corpus rather than as three wholly independent pathways. The NS1–barrier–myeloid unit is best interpreted as a competing-constraint structure in which antiviral pressure, endothelial barrier preservation, and myeloid effector control can become mutually difficult to maintain during progression toward vascular leakage. Severe dengue may emerge when these demands become mutually difficult to satisfy within the same physiological window. This sequence is biologically coherent with known dengue immunopathogenesis, but it is assembled across complementary study types rather than directly observed end-to-end in a single experimental system or single longitudinal cohort.

This coupling is not a causal claim. At the current evidence level, it is best read as a minimum-range organizational interpretation supported by review-anchored evidence, not as causal mechanistic proof: the current corpus is more compatible with a coupled module than with three unrelated recurrent signals, but it does not yet demonstrate that the three components are jointly necessary in a strict mechanistic sense or sufficient as a complete biological account. Within that triad, DENV-M02 is directionally convergent and mechanistically central, but it remains quantitatively thinner than DENV-M01 and DENV-M03 because it is still below the threshold for CI-bearing pooling. The ABM provides C2-level *in silico* support that this candidate coupled set is logically sufficient to produce a synthetic barrier-collapse analog, supporting the theory without elevating it beyond C2.

What these findings exclude at C0 resolution is equally informative: the histamine/PAF axis (rupatadine null) and neuraminidase activity (oseltamivir null) are not rate-limiting in this cascade. Any mechanistic model of severe dengue immunopathogenesis that assigns primary rate-limiting roles to these pathways is not consistent with the C0 evidence.

### 4.2 A minimal competing-constraint model for conceptual integration

**Figure 5:**
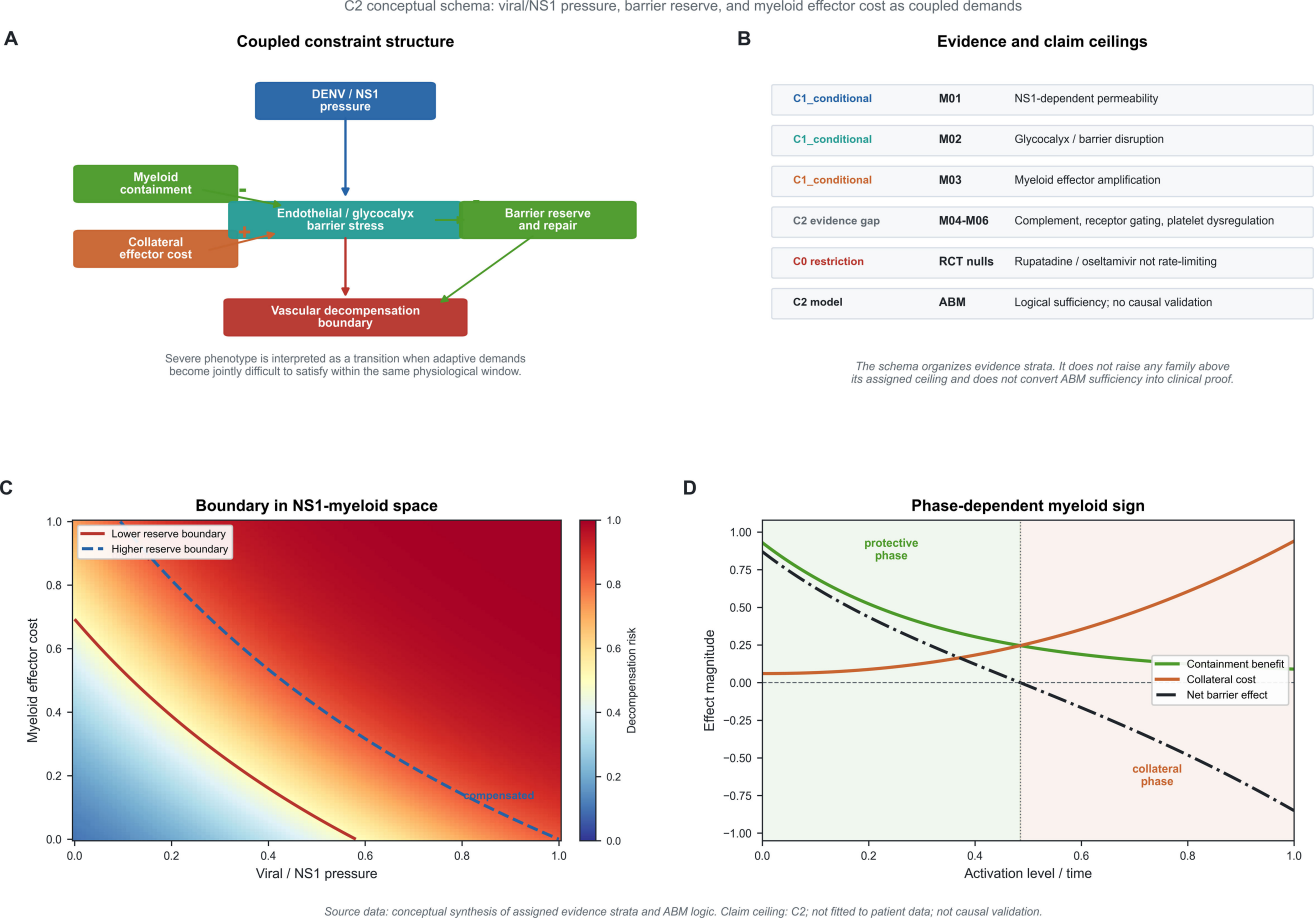
Minimal competing-constraint model of severe dengue vascular decompensation. **(A)** Mechanistic map showing the three competing demands: viral/NS1 pressure (N) propagates downstream as endothelial/glycocalyx stress and barrier injury (injury load L); early myeloid containment (P) reduces effective upstream pressure; late myeloid collateral cost (C) amplifies injury load; barrier reserve (B) and repair capacity (Q) oppose injury load and reduce decompensation risk (R). **(B)** Minimal equations defining the relationships among state variables; symbol table provided. **(C)** Risk landscape (R) as a function of viral/NS1 pressure (x-axis) and myeloid collateral cost (y-axis). Solid red line: decompensation boundary under low barrier reserve. Dashed blue line: boundary under high barrier reserve. Lower barrier reserve shifts the boundary leftwarddownward, making severe outcome accessible under lower stress. **(D)** Phase-dependent myeloid role: early activation is containmentdominant and reduces net barrier risk; sustained or late activation is collateral-cost-dominant and increases net barrier risk. The crossover point represents the transition from protective to damaging myeloid function. Source data: illustrative model; no empirical calibration. This figure is explanatory and conceptual; it is not fitted to patient data, does not validate a causal clinical mechanism, and does not alter the C2 ceiling of the computational component.

To make the competing-constraint logic explicit for immunology and biomedical readers, we present a minimal conceptual model of the NS1–barrier–myeloid account (**Figure 5**). The model represents severe dengue vascular decompensation as the transition point at which residual viral/NS1 pressure and late myeloid collateral cost exceed endothelial/glycocalyx barrier reserve and repair capacity. This model is not fitted to patient data and is not used as causal validation. Its purpose is to clarify the organizational logic that is supported at synthesis level by the systematic review and operationalized at C2 level by the ABM.

The model defines five state variables: viral/NS1 pressure (N), early protective myeloid containment (P), late myeloid collateral cost (C), endothelial/glycocalyx barrier reserve (B), and repair capacity (Q). Effective upstream pressure accounts for early myeloid containment as Neff = max(0, N − cP). Injury load is L = αNeff + βC + γ(N_eff × C), where the interaction term captures the observation that viral/NS1 pressure and late myeloid collateral cost are not additive at high intensities. Barrier reserve is G = B + ρQ. Decompensation risk is R = σ(L − G), where σ is the sigmoid function. Together, these equations express the central claim of the competing-constraint framing: severity arises when injury load exceeds barrier reserve, and that threshold depends simultaneously on all three arms.

The risk landscape (**Figure 5C**) shows that lower barrier reserve shifts the decompensation boundary toward lower values of viral/NS1 pressure and myeloid collateral cost—that is, a fragile barrier makes severe outcome accessible at lower stress on either axis. This motivates the testable hypothesis that reduced endothelial or glycocalyx reserve could shift the decompensation boundary toward lower viral/NS1 pressure or lower myeloid inflammatory cost, a prediction that longitudinal cohort studies with direct barrier measurements could evaluate. The phase-dependent myeloid role (**Figure 5D**) captures the key distinction between early myeloid containment—which reduces effective viral/NS1 pressure and shifts the boundary toward safer parameter space—and late myeloid collateral cost—which amplifies injury load and shifts the system toward decompensation. This is the mechanistic basis for the U3 counter-directional ablation result reported in Section 3.7 and interpreted in detail in Section 4.3 below.

Evidence mapping. Each variable in the minimal model has a direct literature anchor and an ABM counterpart. N (viral/NS1 pressure) is anchored in DENV-M01 and DENV-M02 and corresponds to the viralgrid component in the ABM. C (myeloid collateral cost) is anchored in DENV-M03 (cytokines, sTREM-1, monocyte/macrophage inflammatory amplification) and corresponds to the collateraldamagerate and late immunegrid states. B + Q (barrier reserve and repair) are anchored in DENV-M02 (glycocalyx integrity, HPSE/SDC1 disruption, barrier fragility) and correspond to barrier state and repairrate. P (early myeloid containment) is operationalized in the ABM as the early clearance phase of the immune arm (U3 ablation). A full traceability table mapping each variable to its literature anchor, ABM anchor, current evidence level, and blocked interpretation is provided in **Supplementary File S3** (toymodel_traceability.tsv).

Claim ceiling and limitations. This model is explanatory and conceptual. Its parameters are not fitted to patient data. It does not validate a causal clinical mechanism and does not constitute a predictive clinical tool. Its claim ceiling is C2, identical to the ABM. It does not alter the claim ceiling of any mechanism family and does not constitute a new scientific result beyond the ABM. Its value is pedagogical: it translates the abstract competing-constraint logic into variables and equations that immunology and biomedical readers can interrogate, challenge, and use to formulate testable predictions.

### 4.3 Logical sufficiency vs. causal proof

The ABM U1 battery can be read as four pre-declared falsification-oriented probes of the organizational sufficiency theory. The coupled regime survived all four probes, although U1-B also added an important qualification by showing finite-time path dependence near the boundary rather than simple path independence. These results indicate that the connected-failure regime is a structural property of the coupled mechanism set rather than a single implementation artefact. This constitutes robust *in silico* sufficiency: the three-component coupled module produces the synthetic barrier-collapse analog reliably under varied implementation assumptions.

However, *in silico* sufficiency is not causal proof and does not validate the patient-level mechanism. The ABM makes no empirical claim about specific molecules, specific patients, or specific dose-response relationships. Its claim is organizational: at the level of functional roles (upstream pressure, costly myeloid effector amplification, and finite repair), the coupled set is sufficient to produce the analog of the phenomenon of interest. Three conditions must be met before any claim above C2 is justified: (i) positive quantitative functional data from endothelial perturbation assays (CI-bearing, orthogonal design), (ii) cross-scale correspondence between model components and specific biological measurements, and (iii) patient-linked longitudinal data linking component dynamics to clinical outcome trajectory. None of these is present in the current evidence corpus.

The path-dependence finding (U1-B, max span delta 0.938) demonstrates that within the transition zone, the order in which the viral replication rate crosses the phase boundary matters for the final state of the system. This is an *in silico* property. It does not constitute evidence of clinical hysteresis in dengue, immune memory, or trajectory-locking in patients. The finding supports the plausibility of non-reversible state transitions in barrier-compromise dynamics, but clinical testing of this hypothesis requires longitudinal cohort data with within-patient temporal resolution.

The U2-D, U2-E, and U2-F tests sharpen this interpretation with a three-tier characterization of the model’s sensitivity structure: boundary location is stable under any single form of spatial variation (grain, scale, modest intensity), shows bounded but non-trivial sensitivity only when intensity is pushed to near-maximum values, and in all cases remains more stable than local execution. This structure supports the claim that the transition is a genuine regime property rather than an implementation artefact, while preventing overclaiming of complete invariance.

A fifth probe, the U3 minimality ablation battery, was conducted to ask a narrower question: is the coupled three-arm set not only sufficient but minimal? The battery tested single-arm knockdowns at the boundary-proximal parameter point (VRR = 0.35, RecS=0.50) by setting each arm’s scale to zero in turn across 32 replicates. Two arms proved individually necessary by the pre-declared threshold (delta spanningprobability ≥0.30): the upstream pressure arm (NOUPSTREAM: spanningprobability = 0.0000, delta = 0.4688) and the barrier fragility arm (NOBARRIER: spanningprobability = 0.0000, delta = 0.4688). The myeloid arm was counter-directional: NOMYELOID produced spanningprobability = 1.0000, a delta of −0.5312 relative to the full-model value of 0.4688. The U3 verdict is therefore PARTIALMINIMALITY, not full minimal sufficiency. The counter-directional result for the myeloid arm reveals a structural property of the model’s phase-separated immune architecture: at the boundary parameter point, the myeloid arm serves a dual function—early clearance reduces viral load (dampening spanning) whereas late collateral amplification promotes damage. At VRR = 0.35, clearance dominates: removing the myeloid arm entirely removes clearance, leaving viral replication unchecked, which alone drives spanning. This is biologically coherent with the clinical observation that dengue immunopathology requires a phase transition from containment to dysregulation, but it is strictly an *in silico* property of the current model instantiation and does not constitute evidence that the myeloid effector arm is dispensable in dengue immunopathogenesis.

For immunology interpretation, the ABM should be read as a mesoscale organizational model, not as a fine-grained immune or endothelial simulator. The model omits complement activation, endothelial subtype identity, glycocalyx ultrastructure, platelet-endothelial cross-talk, and myeloid subtype-specific programs. Those omissions are a design choice tied to the model’s organizational purpose.

### 4.4 C0 restrictions as productive constraints

The conventional framing of null RCT findings as negative results obscures their positive epistemic content. In a restriction-first framework, the rupatadine and oseltamivir null findings are among the most valuable pieces of evidence in this review: they definitively exclude specific therapeutic entry points from the mechanistic cascade.

The rupatadine null (RR = 0.68, 95% CI 0.41–1.12) specifically constrains the hypothesis that histamine and platelet-activating factor are primary mediators of NS1-driven plasma leakage. The oseltamivir null (MD =+ 0.1 d, p=0.055) closes the neuraminidase pathway and, in doing so, taxonomically separates dengue barrier disruption from influenza sialic acid-mediated mechanisms. Both constraints are directly compatible with the NS1–HPSE–SDC1/4 axis that constitutes DENV-M02, which does not depend on histamine or neuraminidase activity.

The ABM’s claim ceiling of C2 is also a productive constraint: it delimits what the computational evidence can and cannot authorize, preventing premature mechanistic escalation that would otherwise obscure the genuine evidence gaps identified in the synthesis.

### 4.5 Critical evidence gaps

Four evidence gaps have priority for the next synthesis cycle:

1. DENV-M01 (E3 → E4): The barrier between the current C1conditional ceiling and a C1mechanistic (E4) claim is the absence of CI-bearing quantitative data from a functional intervention study. One independent cohort study reporting Ang-2 AUC with explicit 95% CI would enable the first two-study pool.
2. DENV-M02 (E3 → E4): Functional NS1-specific endothelial barrier intervention data are needed—specifically, dose-response data from HPSE inhibition or SDC1 rescue experiments with CI-bearing outcomes.
3. DENV-M03 (E3 → E4): The sTREM-1 OR = 3.8 (95% CI 1.6–10) remains the single multivariable-adjusted estimate in the corpus. One independent cohort replicating this finding would achieve L3 on the synthesis feasibility ladder and enable a first pooled estimate for myeloid activation severity association—the most achievable next milestone.
4. ABM anchoring (C2 → C1_conditional conditional): The anchor matrix (**Table 2**) specifies that ABM promotion above C2 requires endothelial-pericyte functional data and patient-linked longitudinal evidence linking model component dynamics to clinical outcome trajectory.

### 4.6 Implications for study design: governing the exploratory follow-on

The systematic review and ABM results jointly inform the design of a governed exploratory follow-on study. The three C1_conditional families provide the organizational framework for three functional modules: (i) an upstream-pressure arm testing viral output coherence as an analog of DENV-M01/M02; (ii) a myeloid effector arm testing activation and collateral-cost coherence as an analog of DENV-M03; and (iii) an optional receptor-context arm for exploratory receptor-gating signals (DENV-M04).

The immediate purpose of this follow-on is branch ranking, not mechanism validation. The claim ceiling of this follow-on is C2 (exploratory). Four interpretive restrictions apply, mirroring the C0 constraints from the systematic review: upstream-pressure signal without arm separation is not reported as dengue-specific; generic cytotoxicity is not equated with myeloid effector cost; receptor-context signals without receptor-dependence confirmation are not reported; and no barrier or severity language is used in interpretation.

### 4.7 Limitations

1. Heterogeneous severity definitions. Across the 59 included records, severe dengue was defined using WHO 2009 criteria, WHO 1997 criteria, and study-specific classifications. This heterogeneity limits direct severity-axis comparisons across studies and is acknowledged in the E3 classification for all three C1_conditional families.
2. ABM uncalibrated with bounded heterogeneity sensitivity and deliberate biological abstraction. The ABM was not calibrated to any specific clinical time-series or dose-response dataset. Its parameters define regime boundaries in an exploratory space, not empirically validated operating points. This is by design: the ABM is a logical sufficiency evaluator, not a predictive simulator. The model omits complement activation, endothelial subtype identity, glycocalyx biophysics, platelet-endothelial cross-talk, and myeloid subtype granularity. All boundary-robustness findings are therefore statements about synthetic lattice structure and organizational trade-offs, not patient tissue geometry or fine-grained immune mechanism.
3. NS1 context confusion risk. The ERA research program shares terminology with the dengue program in the glycocalyx-shedding axis (DENV-M02 vs. ERA-M03). Strict enforcement of the dengue-only eligibility policy and explicit documentation of the taxonomic distinction (Section 3.3) prevent contamination within this review.
4. Retrieval bias from unavailable full texts. A total of 38 records flagged for full-text retrieval could not be recovered through open-access channels. This missingness could undercount low-density exploratory families more than the dominant M01–M03 families. Family frequencies and ceiling densities should be read as properties of the recoverable contemporary corpus, not as an exhaustive census.
5. Publication bias. Grey literature was not systematically searched. DENV-M06 (n=1) is particularly vulnerable to publication bias.
6. Language barrier. SCR-00074 (Chinese-language paper, Baicalin/HUVEC/DENV-2) was partially extractable at the qualitative level, but numerical effect sizes remain incomplete due to format and language constraints.
7. Sparse meta-analysis pool. The absence of poolable quantitative estimates (CI available in 17% of M01, 0% of M02, and 8% of M03 records) prevents formal quantitative synthesis. This reflects primary reporting norms in the dengue mechanistic literature.
8. Exclusion of foundational pre-2020 corpus. The *a priori* restriction to 2020–2025 PubMed records was designed to audit the contemporary evidence base. This necessarily excludes foundational dengue mechanistic studies published before 2020. Those studies are cited as background and inform the mechanistic framing but are not treated as claim-bearing synthesis units.

### 4.8 Empirical predictions from the organizational sufficiency theory

Because the theory proposed here is organizational rather than merely descriptive, it generates predictions that could in principle fail. These predictions are not presented as current findings; they are the empirical agenda implied by the present synthesis and ABM.

1. Longitudinal trajectory sensitivity should outperform cross-sectional snapshots. If the U1-B path-dependence result captures a real organizational feature, then serial measurement of upstream NS1/viraemia pressure, endothelial barrier injury markers (SDC1/Ang-2), and myeloid effector proxies (sTREM-1/CXCL10) should separate impending vascular decompensation better than single cross-sectional measurements. Cohorts with longitudinal NS1/viraemia, SDC1/Ang-2, and myeloid-effector proxies should therefore outperform cross-sectional severity studies when testing the transition logic.
2. Myeloid activation may be phase-dependent rather than monotonic. A corollary of the U3 counter-directional ablation result is that myeloid-associated signals may be phase-dependent: early containment-linked myeloid activity may not predict decompensation unless it transitions into a collateral-damage-dominant state. The same myeloid proxy could be protective, neutral, or damaging depending on timing relative to viral/NS1 pressure and barrier fragility.
3. Severity may occupy a boundary region in NS1–barrier–myeloid space, not independent maxima on each axis. The competing-constraint interpretation predicts that severe dengue should be enriched near combinations where antiviral control, endothelial barrier preservation, and myeloid effector regulation become jointly difficult to satisfy. Patients or model systems with extreme values on only one axis should be less informative than trajectories approaching the incompatibility boundary across the coupled space.
4. Single-axis interventions may fail if they do not move the system out of the incompatibility zone. The phase-boundary topology predicts that interventions lowering upstream NS1 pressure before or near boundary crossing should have a larger effect on regime membership than downstream modulation applied after the system has already entered a connected-failure region. The present C0 restrictions already weigh against two specific late-rescue rivals—H1/PAF-focused rescue and neuraminidase-focused rescue—but the broader prediction remains open.

## 5 Conclusion

This systematic review and computational analysis support a minimum-range organizational interpretation of severe dengue vascular decompensation framed as a competing-constraint problem in which antiviral pressure, endothelial barrier preservation, and myeloid effector control can become mutually difficult to maintain during progression toward vascular leakage. In the recoverable 2020–2025 dengue corpus, three mechanistic regularities reach C1_conditional evidence level: NS1-dependent vascular permeability (DENV-M01, n=23), NS1-mediated endothelial glycocalyx disruption (DENV-M02, n=17), and myeloid effector amplification (DENV-M03, n=12). Together, these three families define the minimum-range candidate functional set—the NS1–barrier–myeloid unit—for explaining the transition toward a synthetic barrier-collapse analog in this contemporary evidence base. In parallel, a falsification-tested agent-based model shows that this candidate set is logically sufficient *in silico* to generate that analog. Across the U1 battery, the regime survived four falsification-oriented probes; across the U2 battery, boundary location remained robust to patch scale and recruitment-band expansion, with only bounded sensitivity to very high heterogeneity strength. A subsequent U3 ablation battery showed partial rather than full minimality at the tested boundary point: upstream pressure and barrier fragility were load-bearing, whereas the myeloid arm was counter-directional under the current model architecture.

Two C0 empirical restrictions (rupatadine and oseltamivir null RCTs) constrain the viable therapeutic space and exclude the H1/PAF and neuraminidase axes as rate-limiting steps in dengue vascular immunopathogenesis. Three exploratory mechanisms (DENV-M04, M05, and M06) remain C2 and require replication before any claim escalation. Quantitative meta-analysis is not currently feasible for any family; the minimum viable datasets for first pooled estimates are specified per family and represent the highest-value targets for the next evidence cycle. Future longitudinal cohort studies incorporating NS1/viraemia, endothelial barrier injury markers (SDC1/Ang-2), and myeloid effector proxies, combined with orthogonal functional perturbation assays, represent the required next step for claim escalation beyond C1_conditional.

All findings are bounded within their stated claim levels. The strongest conclusion authorized by this evidence base is as follows: the NS1–barrier–myeloid theory represents a reproducible organizational regularity in the contemporary review corpus and is logically sufficient to produce a synthetic barrier-collapse analog in a falsification-tested computational model. This remains insufficient to constitute a causal mechanistic claim, and the theory should still be understood as a minimum-range organizational account rather than a complete or directly demonstrated triad. Promotion above C1_conditional requires functional intervention data with CI-bearing outcomes from orthogonal experimental designs not present in the current corpus.

## Data Availability

The original contributions presented in the study are included in the article/**Supplementary Material**. Further inquiries can be directed to the corresponding author.

## Supplementary material

Supplementary Table 1 Study-level registry of all 59 claim-bearing included records. Fields: SCR ID, reference number, first author, year, PMID/DOI, study design, population/model, mechanism family, outcome/domain, evidence tier, claim ceiling, reason for inclusion, key signal, and row-level claim note. A machine-readable TSV export and formatted XLSX workbook are provided as supplementary data.

Supplementary Table 2 Excluded records with primary exclusion reason. Fields: SCR ID, title snippet, exclusion category (non-dengue flavivirus/review-no-original-data/indirect-or-method-heavy-noncore/not-retrieved). n=20 excluded + 38 not-retrieved.

Supplementary File S1 ABM parameter sweep outputs in TSV format. Includes the two phase-map tables, U1-A through U1-D outputs, and U2-A through U2-F structural-enrichment sweeps. All files include full parameter configurations and summary metrics per condition.

Supplementary File S2 Systematic review protocol: search strategy (full PubMed query strings), PRISMA 2020 checklist, and PICOS-based meta-analysis eligibility assessment per mechanism family.

Supplementary File S3 Minimal competing-constraint model traceability table (toymodeltraceability.tsv). Maps each model variable to its biological meaning, literature anchor, ABM anchor, current evidence level, claim ceiling, and blocked interpretation. Provided as a machine-readable TSV file.
