# Supplementary material for: Mechanistic constraints in dengue severity: a systematic review with evidence stratification and agent-based evaluation of logical sufficiency
Source: Front Immunol. 2026 Jun 19;17:1831371. doi: 10.3389/fimmu.2026.1831371 (PMC13328458; doi:10.3389/fimmu.2026.1831371)
Supplement: Supplementary File S1 — ABM parameter sweep outputs in TSV format. Includes the two phase-map tables, U1-A through U1-D outputs, and U2-A through U2-F structural-enrichment sweeps. All files include full parameter configurations and summary metrics per condition. [file Supplementaryfile1.zip › 08_Supplementary_File_S2_Protocol_and_Eligibility.docx]

# Supplementary File S2 - Protocol, PRISMA 2020 Checklist, and Meta-analysis Eligibility

This submission-facing supplementary file consolidates the protocol and eligibility materials cited in the manuscript.

## Part A. Systematic Review Protocol

# Mechanistic Constraints in Dengue Severity: A Systematic Review with Evidence Stratification and Agent-Based Evaluation of Logical Sufficiency

## Review type

Systematic review of the contemporary recoverable dengue mechanistic corpus with evidence stratification, claim ceilings, and review-to-model anchoring.

## Scope note

This supplementary protocol describes the executed primary corpus for the present submission freeze. The claim-bearing synthesis set was limited a priori to PubMed/MEDLINE records published between January 2020 and December 2025. Other databases were considered only as possible future sensitivity extensions and were not incorporated into the primary submission corpus reported in the manuscript. Older foundational dengue studies were retained as background citations, not as claim-bearing synthesis units.

## Primary review question

Which dengue host-response mechanisms are most consistently linked to progression toward severe vascular disease, plasma leakage, or shock in the contemporary recoverable corpus, and how do they distribute across vascular permeability, glycocalyx/barrier injury, myeloid effector activation, receptor-context gating, platelet/coagulopathy, and exploratory therapeutic-target families?

## Information source executed

- PubMed/MEDLINE primary search executed
- Search window: January 2020 to December 2025
- No language restriction at search stage; language barriers were documented during post-extraction audit
- Full-text non-retrievals were recorded explicitly and excluded from family assignment

## Eligibility criteria executed

### Include

- Original mechanistic studies in humans, animal models, organoid systems, or cell systems directly relevant to dengue pathogenesis
- Studies involving DENV infection, dengue NS1 exposure, or dengue patient cohorts
- Studies with retrievable quantitative or direction-of-effect information relevant to severity, plasma leakage, endothelial/barrier injury, or myeloid effector activation
- Dengue-relevant interventional trials included as empirical restrictions when they constrained mechanism space

### Exclude

- Reviews, editorials, or corrections without original mechanistic data
- Non-dengue flavivirus studies under the dengue-only eligibility policy
- Original but indirect or method-heavy non-core studies removed from the primary convergence corpus during adjudication
- Records without accessible full text in the executed retrieval workflow

## Screening and adjudication

Title/abstract screening and full-text adjudication were performed by two independent reviewers with consensus resolution. Records not retrievable through the executed access route were retained as documented evidence gaps rather than imputed into mechanism families. A documented adjudication update (2026-03-11) incorporated late-accessed records before synthesis, followed by a final corpus-integrity refreeze (2026-03-14) that removed non-primary or indirect records and corrected study-design miscategorizations before the submission freeze.

## Evidence stratification and claim logic

Included records were assigned to six mechanism families and graded on a five-tier evidence scale (E1-E5). Family-level claim ceilings were then assigned as C0 empirical restriction, C1_conditional robust multi-study regularity, or C2 exploratory. This framework is design-adapted evidence stratification rather than a classical study-by-study risk-of-bias instrument, and it is used to govern what the manuscript is allowed to claim.

## PICOS definition and pooling eligibility criteria

| **PICOS domain** | **Operational definition** |
| --- | --- |
| Population | Dengue patients, DENV/NS1-exposed systems, and dengue-relevant animal, organoid, or cell models; clinical records were required for quantitative pooling. |
| Intervention/exposure | DENV infection, NS1 exposure, mechanistic perturbation, or dengue-relevant therapeutic intervention. |
| Comparator | Non-severe dengue, dengue without warning signs, healthy controls, placebo, unexposed cells/animals, or baseline condition. |
| Outcomes | Vascular permeability, plasma leakage, glycocalyx or endothelial barrier injury, myeloid activation, platelet/coagulation endpoints, and severity-linked quantitative outcomes. |
| Study designs | Clinical cohorts, cross-sectional studies, in vitro systems, animal models, organoid systems, and RCTs for C0 restrictions; pooling required comparable clinical designs. |
| Pooling criterion | At least two records with the same metric, explicit CI or SE, and comparable population/design; otherwise narrative synthesis and meta-analysis readiness auditing were used. |

## Meta-analysis eligibility rule

Quantitative pooling was considered only for DENV-M01, DENV-M02, and DENV-M03, and only when at least two records within a family reported harmonizable outcomes with CI-bearing effect estimates and sufficient design comparability. Under the current freeze, no family met those requirements.

## Part B. PRISMA 2020 Checklist

# Frontiers PRISMA 2020 Checklist

This standalone file is extracted for submission convenience from the Frontiers checklist and manuscript structure.

| **PRISMA item** | **Manuscript location** | **Status** |
| --- | --- | --- |
| Title identifies systematic review | Title page | present |
| Structured abstract | Abstract | present |
| Rationale | Section 1.1-1.3 | present |
| Objectives | Section 1.6 | present |
| Eligibility criteria | Section 2.1.2 | present |
| Information sources | Section 2.1.1 | present |
| Search strategy | Section 2.1.1 | present |
| Selection process | Section 2.1.3 | present |
| Data collection process | Section 2.1.3 | present |
| Outcomes and prioritisation | Section 2.1.4 | present |
| Design-adapted evidence grading / certainty logic | Section 2.1.4 | present |
| Effect measures | Sections 3.2, 3.6, 3.9 | present |
| Synthesis methods | Sections 2.1.5 and 3.9 | present |
| PICOS definition | Section 2.1.5 and Supplementary File S2 | present |
| Reporting-bias assessment | Section 4.6 | present |
| Certainty of evidence | Section 2.1.4 | present |
| Study selection results | Section 3.1 and Figure 1 | present |
| Study characteristics | Table 1 and Supplementary Table S1 | present |
| Individual study results | Sections 3.2-3.6 | present |
| Synthesis of results | Sections 3.2-3.9 | present |
| Discussion | Section 4 | present |
| Limitations | Section 4.6 | present |
| Conclusions | Section 5 | present |
| Registration | PROSPERO field | fill if applicable |
| Protocol availability | Supplementary File S2 | present |
| Funding | Funding section | fill manually |

## Part C. Meta-analysis Eligibility Assessment

# Meta-Analysis Eligibility Assessment - DENV-M01, M02, M03

## Executive verdict

| **Family** | **ID** | **n** | **Clinical studies** | **Hard CI available** | **Poolable records with CI** | **Quantitative MA feasible?** |
| --- | --- | --- | --- | --- | --- | --- |
| Vascular permeability | DENV-M01 | 23 | 13 | 4 | 2 (heterogeneous outcomes) | No |
| Barrier disruption | DENV-M02 | 17 | 6 | 0 | 0 | No |
| Myeloid effector activation | DENV-M03 | 12 | 3 | 1 | 1 (single study) | No |

Conclusion: Quantitative pooling (random-effects or fixed-effect meta-analysis) is not feasible for any of the three C1_conditional families at this baseline. The claim ceiling of C1_conditional (rather than C1) is formally justified by this absence of a CI-bearing pooled estimate.

## 1. PICOS framework applied

| **PICOS element** | **Operationalization** |
| --- | --- |
| P Population | Dengue patients, DENV/NS1-exposed systems, and dengue-relevant animal, organoid, or cell models; clinical records were required for quantitative pooling |
| I Intervention / exposure | DENV infection, NS1 exposure, mechanistic perturbation, or dengue-relevant therapeutic intervention |
| C Comparator | Non-severe dengue, dengue without warning signs, healthy controls, placebo, unexposed cells/animals, or baseline condition |
| O Outcome | Vascular permeability, plasma leakage, glycocalyx or endothelial barrier injury, myeloid activation, platelet/coagulation endpoints, and severity-linked quantitative outcomes |
| S Study design | Clinical cohorts, cross-sectional studies, in vitro systems, animal models, organoid systems, and RCTs for C0 restrictions; pooling required comparable clinical designs |
| Pooling criterion | At least two records with the same metric, explicit CI or SE, and comparable population/design; otherwise narrative synthesis and meta-analysis readiness auditing were used |

## 2. DENV-M01 Vascular permeability (n=23)

### 2.1 Design distribution

| **Design** | **n** | **Pooling eligible?** |
| --- | --- | --- |
| Prospective cohort | 6 | Conditionally |
| RCT | 3 | Conditionally |
| Animal (mouse) | 6 | No |
| Retrospective cohort | 2 | Conditionally |
| Cross-sectional | 2 | Conditionally |
| Cell (endothelial) | 3 | No |
| Case report | 1 | No |

### 2.2 Effect measure distribution

| **Effect measure** | **n** |
| --- | --- |
| not_reported | 16 |
| p-value only | 2 |
| AUC (ROC) | 1 |
| Composite ROC / group difference | 1 |
| RR (null RCT) | 1 |
| Fold-change | 1 |
| Correlation | 1 |

### 2.3 CI availability

- Hard CI (non-FDR, non-omics): 4/23 (17%)
- Records with poolable effect measure + CI: 2/23
- `SCR-00015`: AUC=0.784 (95% CI 0.725-0.835); 3-marker panel (SDC-1+CD163+VCAM-1); single cohort; no comparator study with same panel
- `SCR-00137`: RR=0.68 (95% CI 0.41-1.08, p=0.09); rupatadine null RCT; C0 restriction - cannot pool with observational direction estimates

### 2.4 Pooling blockers

1. Outcome heterogeneity: 23 records use >=15 distinct biomarker endpoints; no two studies share the same primary outcome metric with a CI

2. Design heterogeneity: clinical, in vitro, animal, and case-report evidence cannot be pooled; even within clinical studies, cohort vs. RCT designs address different PICOS

3. Direction of effect: 22/23 (96%) consistent upward direction - supports narrative synthesis, not numeric pooling

4. Missing quantitative reporting: 18/23 records do not report a poolable effect measure; p-only reporting (2 records) is insufficient without SE/CI

5. Null RCT isolation: the two null RCTs (rupatadine, oseltamivir) constrain the therapeutic subdomain of M01 but cannot be pooled with observational marker studies

### 2.5 What IS valid for M01

- Narrative synthesis: 22/23 studies show consistent upward direction of permeability/marker elevation - this is the E3 basis for C1_conditional
- C0 boundary reporting: rupatadine (RR=0.68, 95% CI 0.41-1.12, p=0.09) and oseltamivir (MD=+0.1 day, p=0.055) are formally null and constitute empirical restrictions on therapeutic mechanism claims
- Single-study AUC point estimates: Ang2 AUC 0.97, VEGF AUC 0.99, etc. (SCR-00146) are reportable as exploratory biomarker performance metrics; not pooled
- Promotion pathway: meta-analysis becomes feasible only if >=2 independent cohorts report the same biomarker outcome (e.g., Ang2 severity prediction) with CI using a common threshold or scale

## 3. DENV-M02 Barrier disruption (n=17)

### 3.1 Design distribution

| **Design** | **n** | **Pooling eligible?** |
| --- | --- | --- |
| Animal (mouse) | 8 | No |
| Prospective cohort | 5 | Conditionally |
| Cell (endothelial) | 3 | No |
| Cross-sectional | 1 | Conditionally |

### 3.2 Effect measure distribution

| **Effect measure** | **n** |
| --- | --- |
| not_reported | 10 |
| p-value only | 3 |
| OR | 1 (CI not extracted) |
| RR | 1 (CI not reported) |
| Fold-change | 1 |
| Qualitative domain swap | 1 |

### 3.3 CI availability

- Hard CI (non-FDR): 0/17 (0%)
- Records with poolable effect measure + CI: 0/17
- `SCR-00052`: OR=2.5 (ANGPTL4 + plasma leakage) - CI field shows "not_extracted"; even if extracted this is a single study
- `SCR-00072`: RR not reported; CI not reported
- `SCR-00074`: P<0.05-0.01 (in vitro baicalin); not clinical; not poolable

### 3.4 Pooling blockers

1. Zero poolable records: no record in M02 meets the minimum standard of [clinical study + effect measure + CI] after the corpus-integrity refreeze

2. High in vitro and animal dominance: 12/17 records (71%) are non-clinical by design

3. Mechanistic heterogeneity within family: NS1-HPSE-SDC1/4 axis, TJ disruption, ANGPTL4-Ang1/2, autophagy modulation, structural NS1 determinants - these are convergent mechanistically but cannot be pooled as a single outcome

4. Direction of effect: 15/17 (88%) upward consistent - supports E3 mechanistic narrative, not pooling

### 3.5 What IS valid for M02

- Narrative mechanistic synthesis: NS1-HPSE-SDC1/SDC4 axis confirmed across animal + human + in vitro; anti-NS1 mAb TEER rescue is the strongest functional piece
- Structural evidence (SCR-00126): GDI residues 91-93 required for DENV NS1-induced vascular leak - this is a mechanistic constraint, not poolable as a risk estimate
- ANGPTL4 (SCR-00052): OR=2.5 is a single-study point estimate; extracting the CI is the minimum prerequisite for its individual reporting
- Promotion pathway: feasible only if ANGPTL4, SDC-1, or HPSE studies in clinical cohorts begin reporting adjusted ORs with CI

## 4. DENV-M03 Myeloid effector activation (n=12)

### 4.1 Design distribution

| **Design** | **n** | **Pooling eligible?** |
| --- | --- | --- |
| Animal (mouse) | 4 | No |
| Cell (endothelial) | 3 | No |
| Cell (myeloid) | 2 | No |
| Prospective cohort | 2 | Yes |
| Cross-sectional | 1 | Yes |

### 4.2 Effect measure distribution

| **Effect measure** | **n** |
| --- | --- |
| not_reported | 7 |
| fold_change | 2 |
| p-value only | 2 |
| OR | 1 |

### 4.3 CI availability

- Hard CI: 1/12 (8%)
- Poolable with CI: 1/12
- `SCR-00037`: sTREM-1 >= 130 pg/mL -> hemoconcentration; OR=3.8 (95% CI 1.6-10; p=0.0020); logistic regression adjusted; single study

### 4.4 Duplicate records

- `SCR-00097` is a duplicate PDF of `SCR-00080` (both: Wong et al. DENV NS1 caspase-1 NLRP3-independent IL-1beta); duplicate does not add independent evidence

### 4.5 Pooling blockers

1. Single poolable record: sTREM-1 OR=3.8 is an isolated finding from one prospective cohort; cannot pool without >=1 independent replication

2. Non-clinical majority: 9/12 records are in vitro, animal, or cell-system studies; the 3 clinical records use different outcomes (OR vs. qualitative NETs vs. transcriptomic subset profiling)

3. Duplicate PDF reduces effective n: effective unique clinical studies = 11, not 13

### 4.6 What IS valid for M03

- sTREM-1 point estimate: OR=3.8 (95% CI 1.6-10) is the strongest single quantitative finding in the M01-M03 group; reportable as an individual study result
- AXL-monocyte tropism: supported by in vitro evidence but not yet replicated in clinical cohorts
- Direction: 12/12 (100%) consistent upward - the strongest direction signal in the three families
- Promotion pathway: a second independent cohort reporting sTREM-1 with an adjusted OR and CI would enable a 2-study fixed-effect pool - the most achievable next step in the entire evidence base

## 5. Cross-cutting barriers to pooling

| **Barrier** | **M01** | **M02** | **M03** |
| --- | --- | --- | --- |
| Outcome heterogeneity (>=10 distinct endpoints) | yes | yes | yes |
| Missing CI (>80% of records) | yes | yes | yes |
| Mixed clinical + non-clinical designs | yes | yes | yes |
| No two studies share same primary outcome with CI | yes | yes | yes |
| p-only reporting dominates | yes | yes | yes |
| Functional / mechanistic evidence not poolable | - | yes | yes |
| Duplicate PDF records | - | - | yes |

## 6. Synthesis feasibility ladder

| **Level** | **Requirement** | **M01** | **M02** | **M03** |
| --- | --- | --- | --- | --- |
| L1 Narrative direction | >=2 studies consistent direction | yes (22/23) | yes (15/17) | yes (12/12) |
| L2 Point estimate reporting | >=1 study with effect measure + CI | yes (2) | no | yes (1) |
| L3 Two-study pool | >=2 independent studies, same outcome, CI | no | no | no |
| L4 Random-effects MA | >=3 studies, same PICOS, CI | no | no | no |
| L5 Adjusted MA | >=3 studies, adjusted effect + CI | no | no | no |

Current maximum achievable: M01 = L2, M02 = L1, M03 = L2

## 7. Action requirements for future eligibility

### Minimum viable dataset for a 2-study pool (L3)

| **Family** | **Target outcome** | **Required addition** |
| --- | --- | --- |
| DENV-M01 | Ang2 severity prediction (AUC / ROC with CI) | 1 independent cohort reporting Ang2 AUC with 95% CI and common threshold |
| DENV-M02 | ANGPTL4 plasma leakage (OR with CI) | Extract CI from SCR-00052 + 1 independent cohort |
| DENV-M03 | sTREM-1 hemoconcentration (OR with CI) | 1 independent cohort replicating sTREM-1 >= 130 pg/mL OR with CI |

### Minimum viable dataset for a 3-study random-effects pool (L4)

All three families require 2 additional independent clinical cohort studies reporting the same outcome metric with CI.

## 8. Governance integration

- Claim ceiling justification confirmed: C1_conditional (not C1) is formally correct for all three families - a CI-bearing pooled estimate does not exist
- C0 restrictions are not poolable: the two null RCTs in M01 constrain the therapeutic space; they should not be pooled with the observational direction-of-effect evidence
- sTREM-1 OR=3.8 (M03): strongest isolated quantitative result in the corpus; should be reported in Results as a single-study finding with explicit label "single study, no independent replication"
- Do not declare convergence as equivalence to pooled effect: consistent direction (L1) across 13-30 studies is the basis for E3; it does not substitute for a quantitative pooled estimate
